# Investigating the effects of genetic risk of schizophrenia on behavioural traits

**DOI:** 10.1038/s41537-020-00131-2

**Published:** 2021-01-22

**Authors:** Adam Socrates, Jessye Maxwell, Kylie P. Glanville, Marta Di Forti, Robin M. Murray, Evangelos Vassos, Paul F. O’Reilly

**Affiliations:** 1 SGDP Centre, Institute of Psychiatry, Psychology and Neuroscience, King’s College London, London, UK; 2 Institute of Psychiatry, Psychology and Neuroscience, King’s College London, London, UK; 3 Department of Genetics and Genomic Sciences, Icahn School of Medicine at Mount Sinai, New York City, New York USA

**Keywords:** Schizophrenia, Biomarkers

## Abstract

To characterise the trait-effects of increased genetic risk for schizophrenia, and highlight potential risk mediators, we test the association between schizophrenia polygenic risk scores (PRSs) and 529 behavioural traits (personality, psychological, lifestyle, nutritional) in the UK Biobank. Our primary analysis is performed on individuals aged 38–71 with no history of schizophrenia or related disorders, allowing us to report the effects of schizophrenia genetic risk in the sub-clinical general population. Higher schizophrenia PRSs were associated with a range of traits, including lower verbal-numerical reasoning (*P* = 6 × 10^–61^), higher nervous feelings (*P* = 1 × 10^−46^) and higher self-reported risk-taking (*P* = 3 × 10^−38^). We follow-up the risk-taking association, hypothesising that the association may be due to a genetic propensity for risk-taking leading to greater migration, urbanicity or drug-taking — reported environmental risk factors for schizophrenia, and all positively associated with risk-taking in these data. Next, to identify potential disorder or medication effects, we compare the PRS–trait associations in the general population to the trait values in 599 medicated and non-medicated individuals diagnosed with schizophrenia in the biobank. This analysis highlights, for example, levels of BMI, physical activity and risk-taking in cases in the opposite directions than expected from the PRS–trait associations in the general population. Our analyses offer simple yet potentially revealing insights into the possible causes of observed trait–disorder associations, which can complement approaches such as Mendelian Randomisation. While we urge caution in causal interpretations in PRS cross-trait studies that are highly powered to detect weak horizontal pleiotropy or population structure, we propose that well-designed polygenic score analyses have the potential to highlight modifiable risk factors that lie on the path between genetic risk and disorder.

## Introduction

While much is known about behavioural traits associated with schizophrenia diagnosis and medication^1^, and prospective studies have provided insights into behavioural traits common during the years leading to diagnosis^2^, less is known about behavioural traits associated with elevated genetic risk for schizophrenia in the sub-clinical general population. The latter could be useful for highlighting potential mediators of disorder risk because their study should be less affected by the confounding effects of medication or disorder pathophysiology, which may have pre-clinical onset.

Modification of lifestyle or behavioural risk factors has the potential of reducing disorder risk before the initiation of pathology. However, identifying such risk factors using clinical trials is typically infeasible and from standard observational data is highly challenging due to the complex network of traits that generates a myriad of non-causal associations^3^. The unidirectional nature of genetic effects on traits, however, provides a convenient ‘anchor’ to build more powerful causal inference from observational data^4^.

Polygenic risk scores (PRSs), which correspond to a weak proxy of an individual’s genetic liability to a trait or disorder, can be used to infer genetic overlap between phenotypes via predicting one phenotype from the PRS of another^5–9^. Identifying overlap between genetic risk for a disorder and a trait can be useful both to characterise the trait–PRS associations and in prioritising putative disorder-modifying traits because genetic risk for traits that *do* modify disorder risk must form a sub-component of genetic risk for the disorder itself. Once genetic overlap has been established, the next step is to infer which of the following is most likely responsible: horizontal pleiotropy (i.e. the trait is non-causal of the disorder), vertical pleiotropy (the trait affects the disorder or vice versa) or that the observed overlap is an artefact of population structure or ascertainment^5,10^.

Several studies have tested for genetic overlap between schizophrenia and a small number of putative risk factors, such as cognitive, smoking and other psychiatric traits^11–13^, while one study has also tested for associations between schizophrenia genetic risk and a large number of traits in the UK Biobank^9^. However, while these studies have used techniques such as Mendelian Randomisation^9^ and Genomic SEM^13^ to infer the causal relationships underlying the observed genetic overlap, they have not separated out non-diagnosed from diagnosed — medicated and non-medicated — samples, which may have compromised inferences made. Here we analyse these individuals separately. We utilise the large sub-cohort of the UK Biobank with no history of schizophrenia (or related disorders) in an exploratory study to test for the effects of schizophrenia genetic liability in the general population, and independently evaluate the large number of schizophrenia medicated and non-medicated cases; all of these individuals were collected and assayed as part of the UK Biobank in the same way. Contrasting the results across these groups provides a novel way of gaining insights that can help to distinguish between vertical and horizontal pleiotropy, as well as between disorder and medication effects. We also perform sensitivity testing to investigate potential confounding by population structure. To demonstrate how these results can motivate further interrogation of specific findings, we follow-up the positive association between schizophrenia PRS and risk-taking, investigating whether a greater propensity for risk-taking may expose individuals to modifiable risk factors for schizophrenia, such as illicit drug use.

Our analyses should be considered an initial exploratory attempt to identify behaviours that may mediate risk of schizophrenia. We consider the simplicity of our approach to be a strength as an exploratory analysis, highlighting and contrasting effects in non-psychiatric, medicated and non-medicated cases in the same cohort in a direct and transparent way, making few assumptions. Analyses of this type could act as a complement to other more technical approaches, such as Genomic SEM or Mendelian Randomisation, for investigating causality in trait–disorder associations^4^.

## Results

### Polygenic risk score prediction of behavioural traits

We performed PRS analyses (see Methods) using *PRSice-2*^14^, with the schizophrenia GWAS summary statistics from the Psychiatric Genomics Consortium (PGC) as base data^15^ and the UK Biobank data on 307,823 individuals with no history of schizophrenia, bipolar disorder or major depressive disorder as target data (see Methods, Supplementary Tables 1 and 2 for traits and sample sizes). Associations between the most predictive schizophrenia PRS and the 529 behavioural traits from the UK Biobank were tested using linear and logistic regressions in *PRSice-2*, controlling for age, sex, Townsend deprivation index and the first 15 principal components (PCs) to control for population stratification. We set a highly conservative significance threshold of *P* < 1 × 10^−7^ based on PRS including SNPs at a threshold of *P*_T_ < 0.05 (the best-fit schizophrenia PRS in leave-one-out analyses^15^) across 529 target behavioural traits. This stringent threshold was used to minimise significant hits being due to chance. Schizophrenia PRS showed significant (*P* < 1 × 10^−7^) associations in 104 of the 529 behavioural traits in the screened sub-cohort of 307,823 individuals. Phenotypic correlations between these traits can be seen in Supplementary Figs. 1 and 2. To verify the reliability of these polygenic associations, we repeated the analyses using PRS comprising only the 108 sentinel genome-wide significant schizophrenia variants^15^, based on the assumption that the effect size estimates of genome-wide significant variants should be less affected by population structure than non-significant variants; 77 of the 104 associated traits were nominally significant (*P* < 0.05) based on the PRS comprising only genome-wide significant variants. The results of all 104 significant associations are displayed in Supplementary Table 2.

**Fig. 1:**
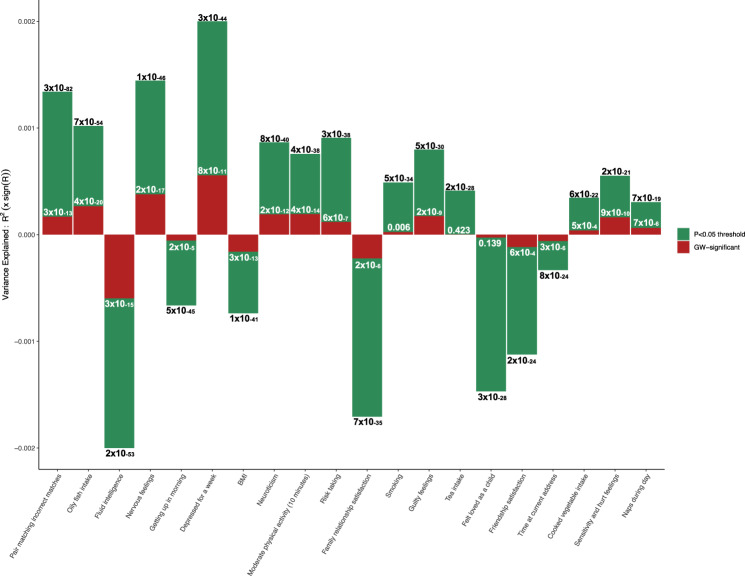
Bar plot showing 20 of the most significant associations. *y*-axis shows target trait variance explained, upwards for positive and downwards for negative associations; with *P*-values of association shown on bars. The green bars show the best-fit schizophrenia PRS and the corresponding target traits (*x*-axis), and the red bars show the association results when using the genome-wide significant (GW-significant) schizophrenia PRS instead (based on the 108 sentinel schizophrenia SNPs). See Supplementary Table 2 for details and to inspect the selection of these 20 results among the top results.

Figure 1 illustrates 20 of the most significant associations, with highly related traits omitted (see Supplementary Table 2), showing both the results based on the best-fit PRS (green) and the PRS calculated from only the 108 genome-wide significant schizophrenia SNPs (red). The markedly higher predictive power of the best-fit PRS is apparent, yet the PRS–trait associations do not appear to be due merely to the inclusion of non-significant SNPs given the consistency with the GW-significant PRS results. Reassuringly, the significant association with tea intake is non-significant when tested using the GW-significant PRS, although the association with cooked vegetable intake remains significant. We consider how horizontal pleiotropy or population structure may be responsible for these unexpected top-ranking associations in the final sub-section of the Results, but first focus on the findings that may indicate plausible targets of intervention. It is noticeable, though perhaps unsurprising given the deleterious nature of even prodromal stage schizophrenia^2,16^, that most of the associations are in a direction that reflects ‘negative trait’ outcomes with higher genetic risk for schizophrenia; for example, positive correlations with nervous (*P* = 1 × 10^−46^) and guilty (5 × 10^−30^) feelings, and negative correlations with fluid intelligence (*P* = 2 × 10^−53^) and friendship satisfaction (*P* = 2 × 10^−24^). Since these analyses have been performed on a sample of individuals with no history of schizophrenia, then they should reflect the effects of genetic risk for schizophrenia in the general unaffected population. We test this claim further in the next sub-section.

### Contrasting trends in the general population and diagnosed samples

The presence of individuals with schizophrenia in the UK Biobank allows us to contrast the PRS-by-trait trends observed in the undiagnosed general population with values of the corresponding traits in diagnosed individuals. There are 599 individuals in the full genotyped UK Biobank data with an ICD-10 diagnosis (from Hospital Episodes Statistics) of ‘schizophrenia, unspecified’. This is the largest category of official schizophrenia diagnosis in the UK Biobank. Of these individuals, 297 reported presently taking antipsychotic treatment during the baseline verbal interview, which is also when most traits tested here were measured; however, many traits were measured as part of the Mental Health Questionnaire performed approximately 8 years after baseline interviews and so there will be greater misclassification of ongoing-treatment status in relation to these traits and correspondingly smaller estimated effects (Supplementary Table 2). There is also likely to be healthy participation bias, in which diagnosed individuals participating in the study are likely to be less severe than diagnosed individuals in the broader population. Furthermore, diagnoses correspond to lifetime diagnoses, whereas medication status corresponds to that at baseline. Therefore, diagnosed non-medicated individuals may have comparatively lower severity on average, and so differences between the general population and non-medicated cases may be underestimated, while differences between medicated and non-medicated cases may be over-estimated. Finally, since the number of individuals with schizophrenia in these data is relatively small, in particular those on medication, then these findings should be considered with caution.

**Fig. 2:**
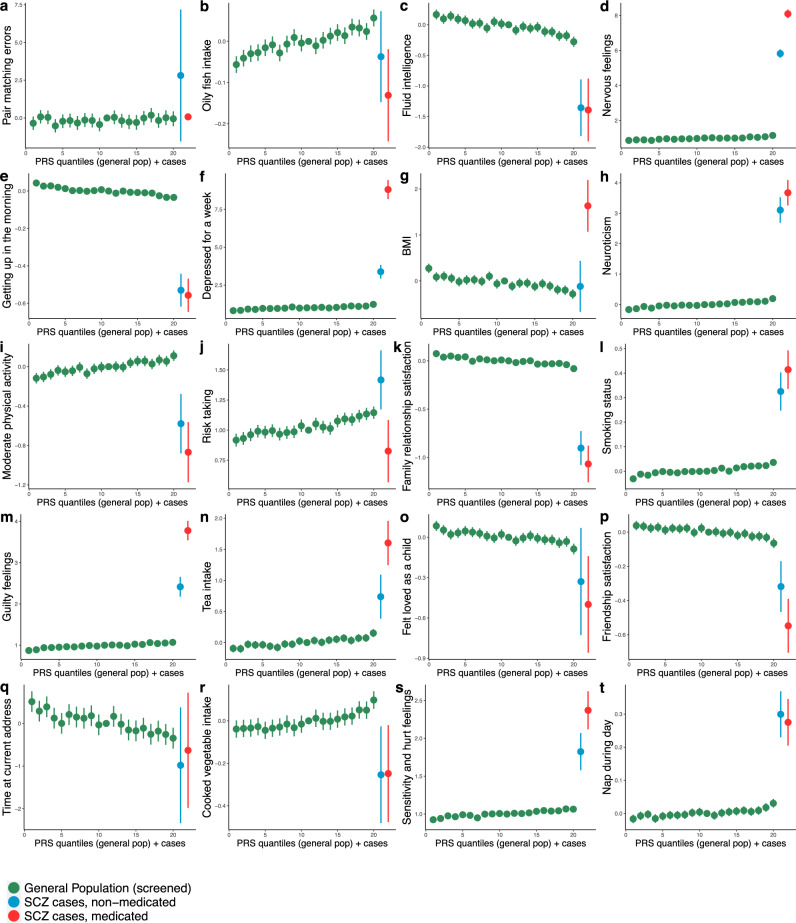
PRS-by-trait quantile plots (in green) indicating the trends of association between the best-fit schizophrenia PRS and the target behavioural traits among 20 of the most significant associations, matching those of Fig. 1. The quantile plot is ordered along the *x*-axis from low to high genetic risk for schizophrenia, according to PRS in the target (UK Biobank) data, with each green point representing the average target trait value of a 5% quantile of the sample. The *y*-axis shows the regression coefficients corresponding to the effects of the PRS on the trait, controlled for covariates (see Methods). Non-medicated (blue) and medicated (red), at baseline, diagnosed individuals are appended to the right end of each plot, reflecting the expected higher genetic burden of diagnosed individuals compared to unaffected individuals (see Main Text). Vertical lines represent 95% confidence intervals; these appear absent for some traits with a large range and are larger in the two categories of cases due to their smaller sample sizes.

Figure 2 displays the PRS-by-trait trends in the undiagnosed general population as quantile plots (in green), with the trait values for non-medicated (blue) and medicated (red) schizophrenia cases appended to the right end of the *x*-axes. Depicting the cases as having the highest genetic liability is appropriate because, on average, cases are expected to have a higher genetic liability than unaffected individuals, and the low predictive power of the PRS ensures that even individuals in the top 5% quantile of the undiagnosed sub-cohort will only have a moderately elevated schizophrenia risk on average. The cases’ PRS fall between 20^th^ out of 21^st^ quantiles, and medicated cases have higher PRS on average compared to non-medicated cases (see Supplementary Figs. 3 and 4).

The plots in Fig. 2 correspond to the traits of Fig. 1 and are also shown in order of association significance (notice the different *y*-axis scales when comparing the trends).

In 13 of the 20 results shown in Fig. 2, the trait values in schizophrenia cases are in the direction expected according to the PRS-by-trait trend, and in 12 of these 13, the medicated cases have a more extreme trait value. For example, in Fig. 2d we observe higher levels of nervous feelings in undiagnosed individuals with higher genetic risk for schizophrenia, even higher levels in non-medicated schizophrenia cases, and the highest levels of nervous feelings in medicated schizophrenia cases. The trends towards the case trait values observed with increasing genetic risk for schizophrenia in the general population could be caused by: (i) the trait being a (modifiable) risk factor for schizophrenia (vertical pleiotropy), (ii) the pre-clinical or spectrum-like manifestation of the disorder in the general population, which essentially constitutes effects downstream of risk factors (horizontal pleiotropy), or (iii) misclassification of schizophrenia cases as being unaffected. The alternative explanations (i) and (ii) cannot be easily distinguished without specific follow-up studies (see next sub-section). To elaborate on (iii), if misclassification of schizophrenia cases as unaffected is more common among individuals with higher schizophrenia PRS, then a downstream effect of schizophrenia on, e.g. nervous feelings may have generated the observed PRS-by-trait positive trend. However, if such misclassification does exist, then it may play a small role in the observed results since almost half (7 of 20) show discordant results between the general and affected sub-cohorts.

The fact that medicated cases generally have more extreme trait values than non-medicated cases could be explained by medicated cases having greater severity of disorder than non-medicated cases.

However, some of the results show little difference between the groups or the reverse trend, which may be indicative of medication effects. For instance, while BMI reduces with increasing schizophrenia PRS (Fig. 2g), medicated cases have markedly higher BMI compared to the general population (*P* = 4.9 × 10^−18^; see Supplementary Table 2) and non-medicated cases, which is consistent with the known side-effect of antipsychotic medication of increased BMI. Figure 2j may point to some behavioural explanation for this, since higher schizophrenia PRS is associated with higher physical activity, while medicated individuals show the lowest levels of activity. Negative symptoms such as apathy and lack of motivation may also contribute to lower physical activity in people with schizophrenia, as well as side-effects of antipsychotic medication, lack of self-efficacy and reduced capacity to prioritise health benefits, which may not be present in the general population^17^. There is increased self-reported risk-taking with higher schizophrenia PRS (Fig. 2j), but individuals on medication have lower levels of risk-taking than those not on medication (*P* = 3 × 10^−3^). Interestingly, dopamine agonist drugs have been found to increase risky behaviours in several studies on Parkinson’s disease patients^18,19^, and so these results, pertaining to antipsychotic-induced dopamine blockade, may reflect the reverse effect.

Supplementary Figs. 5–8 display similar plots for the remaining top 80 PRS-by-trait association results with *P* < 1 × 10^−7^, and Supplementary Table 2 provides results of testing the difference between trait values in schizophrenia cases compared to those of the top quantile in the unaffected general population for all 104 significantly associated traits.

The widespread significant and substantial differences observed in the behavioural traits between schizophrenia cases and the undiagnosed individuals (Fig. 2, Supplementary Table 2 and Supplementary Figs. 5–8): (1) supports the recorded diagnosis of schizophrenia (i.e. that these individuals behave differently from the general population) and (2) stresses the need to perform PRS cross-trait analyses, and causal inference analyses from genetic data, that are stratified by disorder/medication status.

### A possible path to schizophrenia via the genetics of risk-taking

Here we follow-up one of our top-ranking associations that purporting to self-reported risk-taking (response to question ‘Would you describe yourself as someone who takes risks?’), since we consider this a plausible target for therapeutic intervention. We also perform this follow-up analysis to highlight the potential for our results in hypothesis-generation and to show how specific results can be further interrogated to gain greater insights into their underlying causes.

We hypothesise that a part of the aetiology of schizophrenia may derive from a genetic propensity for risk-taking, resulting in greater exposure to drug-taking, migration or urbanicity — reported risk factors for schizophrenia^20–23^. Testing these pathways explicitly is underpowered here due to the relatively low power of a UK Biobank risk-taking PRS and the limited number of schizophrenia cases for such analyses, so instead we tested the phenotypic associations and those with the schizophrenia PRS. Migration patterns were characterised in three ways: Euclidean distance moved between birth and current residence, birth and current residence population density (and their difference) and time spent at current residence (see Methods). We also tested associations with several ‘control traits’, such as ‘leg pain on walking’, ‘breast fed’ and ‘month attended baseline assessment centre’ (see Methods) for comparison, since epidemiological testing on such large sample sizes are vulnerable to producing highly significant associations that are the consequence of small biases or statistical artefacts.

Testing in the undiagnosed individuals, self-reported risk-taking is positively associated with lifetime distance moved (*P* = 3 × 10^−123^; *r*^*2*^ = 0.001) and change in population density (*P* = 7 × 10^−37^; *r*^*2*^ = 0.0004), and is negatively associated with time spent in current residence (*P* < 1 × 10^−300^; *r*^*2*^ = 0.007). Risk-taking is also positively associated with self-reported substance abuse (*P* = 8 × 10^−74^; *r*^*2*^ = 0.008). While these results are consistent with the proposed pathway (see Supplementary Table 3 for all results), we caution against their strong interpretation because of their small explanatory power, their attenuation when also controlling for Townsend deprivation index and education level, the sensitivity of small *P*-values to stochastic variation^24^, and because the associations with many of the control traits were also significant.

Next, we tested for the association between schizophrenia PRS and these traits in the undiagnosed sub-cohort (see Supplementary Table 4). With the exception of left-hand grip strength, blood pressure device ID (i.e. type of blood pressure measuring device) and population density of birth place, all the nominally significant results related to the migration variables and substance use and all the non-significant results corresponded to the control traits (threshold of *P* < 0.05 for this sub-analysis, though we caution discrete interpretation of *P*-values in general^24,25^). Schizophrenia PRS was positively associated with population density of current residence (*P* = 3 × 10^−11^), self-reported substance abuse (4 × 10^−9^), ever-smoked-cannabis (7 × 10^−8^) and change in population density from birth to current residence (*P* = 6 × 10^−3^), and negatively associated with time spent at current residence (*P* = 1 × 10^−21^) and distance travelled (5 × 10^−4^). However, each association is attenuated when Townsend deprivation index and education are controlled for, especially lifetime change in population density. This likely reflects a complex network of mediating, pleiotropic and causal relationships between the genetics of risk-taking, education, socio-economic status, migration and drug-taking patterns, which could generate cross-generational effects such as the association observed between schizophrenia PRS and birthplace population density. Focussed follow-up studies will be required to further unravel the proposed risk-taking–schizophrenia pathway in order to consider it a possible target for intervention.

### Potential confounding in PRS–trait associations

**Fig. 3:**
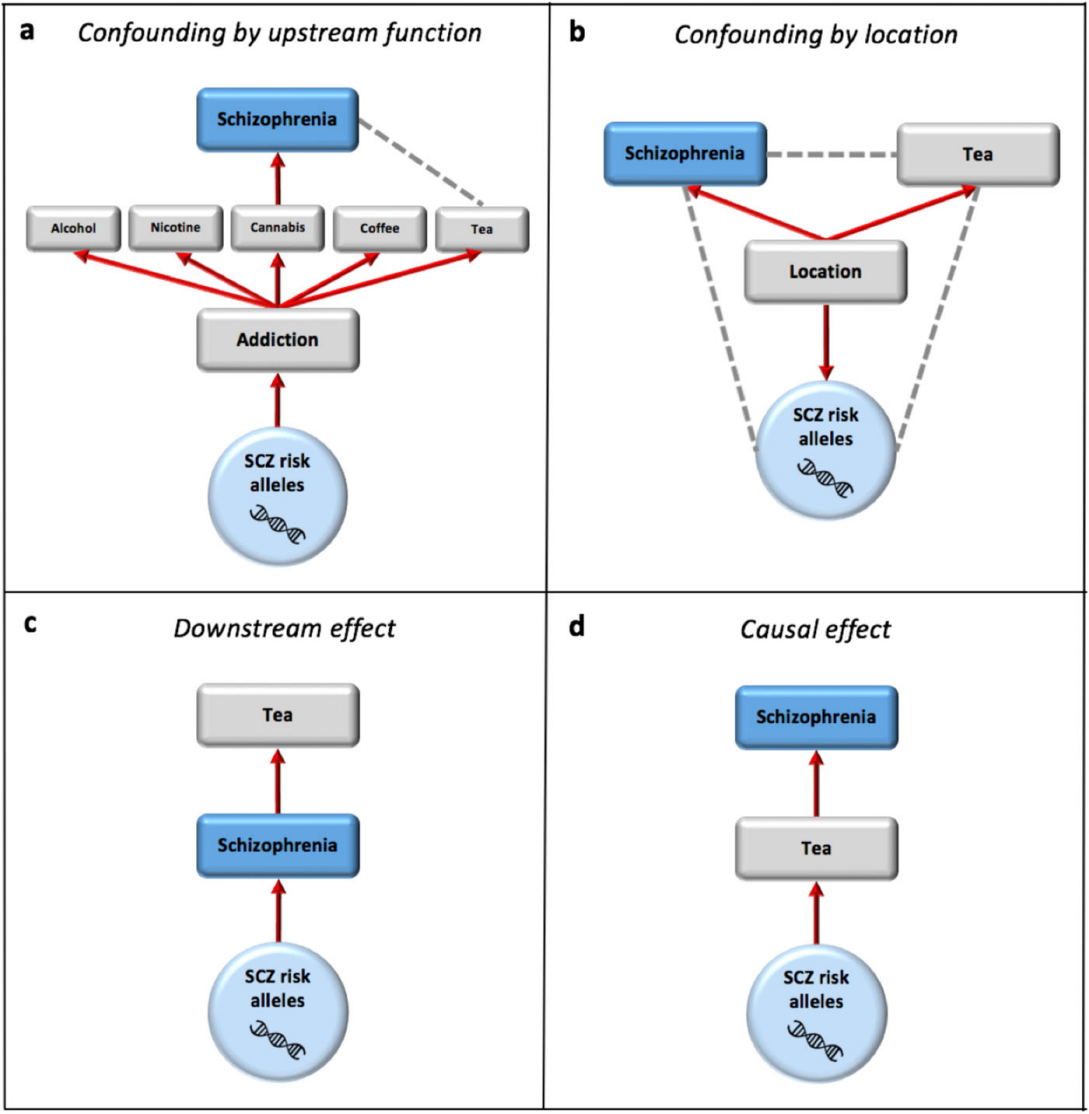
Four possible causal explanations for the schizophrenia-PRS vs tea drinking association. Red arrows reflect causal paths and grey dotted lines depict confounded associations induced by the causal relationships. schizophrenia ‘risk alleles’ refer to those included in the PRS, thus comprising genuine schizophrenia risk alleles (/risk haplotypes) and those with no effect on schizophrenia. **a** Some fraction of schizophrenia risk alleles are addiction risk alleles, which influence addiction to multiple substances. The causal relationship between cannabis and schizophrenia assumed here induces the association between schizophrenia-PRS and tea consumption, confounded-by-function (addiction here, used for illustration only); **b** schizophrenia risk allele frequencies vary with location (due to genetic drift, etc.) and so too do environmental factors influencing tea consumption and schizophrenia risk, inducing correlations between all three; **c** schizophrenia risk alleles increase risk for schizophrenia. Schizophrenia increases tea consumption; **d** schizophrenia risk alleles increase tea consumption. Tea consumption increases risk for schizophrenia.

While many of the associations in Fig. 1 have been implicated in schizophrenia aetiology previously^20^, there are also highly significant unexpected associations between the best-fit schizophrenia PRS and tea intake (*P* = 1.7 × 10^−28^) and cooked vegetable intake (*P* = 6.4 × 10^−22^). While the association between the GW-significant PRS and tea intake is reassuringly non-significant (*P* = 0.42), it remains a concern for the many similar analyses being conducted in the field^6–9^ that there is such a significant association between the best-fit PRS and a trait that is expected to play no causal role in schizophrenia. We exploit the engaging and memorable nature of this example to highlight the potential for confounding to generate PRS–trait associations, either in the form of horizontal pleiotropy or due to population structure. In Fig. 3, we illustrate what we consider to be four of the main broad causal explanations for PRS–trait associations, using the association with tea intake as an example, and consider how each can be further tested.

In Fig. 3a we illustrate how ‘horizontal pleiotropy’ may typically arise, via the multiple downstream effects of a causal risk factor or function encoded by the genome (here addiction, leading to the putatively causal factor cannabis smoking). This (hypothetical) relationship would result in the genetic risk for schizophrenia being associated with tea intake. This relationship could be tested by controlling for cannabis smoking, but relatively few unaffected individuals in the UK Biobank answered the questions on cannabis smoking. There are positive associations phenotypically between tea drinking and cannabis smoking (*P* = 6 × 10^−4^; maximum frequency of cannabis smoking), and also substance abuse (*P* = 0.05), but alcohol consumption (*P* = 3 × 10^−106^) and coffee consumption (*P* < 1 × 10^−308^) are negatively correlated with tea drinking. The best-fit schizophrenia PRS is positively associated with current smoking status (*P* = 2 × 10^−37^) and ever-taken-cannabis (*P* = 7 × 10^−17^), consistent with the literature^26,27^, but is negatively associated with beer intake (*P* = 2 × 10^−11^) and coffee intake (*P* = 3 × 10^−12^). Thus, any link with propensity to addiction is not straightforward and unlikely to be explained by the simple relationship in Fig. 3a.

The potential for confounding by location (Fig. 3b) in polygenic score analyses has been highlighted in several recent publications^5,28^. We repeated the analyses extending the number of PCs adjusted for from 15 to 40 PCs and observed little change in results (Supplementary Fig. 9). While confounding by location is, thus, perhaps unlikely to be the main explanation for the association with tea drinking, and the results in general, the large sample size here means that even subtle confounding effects — not well-captured by top PCs — could be responsible for highly significant associations, and so without a thorough investigation of potential confounding by population structure this possibility cannot be ruled out.

While individuals with schizophrenia may consume more tea (Fig. 3c) — due to spending more time indoors, for example — our analyses were performed in the unaffected sub-cohort and so the observed association should not be due to disorder onset. Misclassification of cases as being unaffected could have caused the association, although our results overall indicate that such an effect may be small (see previous section).

We believe that tea being a causal risk factor for schizophrenia (Fig. 3d) is the least likely of these potential explanations. Bidirectional Mendelian Randomisation^29^ and the Latent Causal Variable approach^30^ can be applied to distinguish between causal effects in either direction (Fig. 3c, d), but distinguishing these from confounding-by-function (Fig. 3a) is extremely challenging without highly rich or prospective data (note that methods removing variants displaying horizontal pleiotropy would likely rule-out cannabis as a causal factor in the scenario of Fig. 3a).

Each of the models in Fig. 3 are severe simplifications of reality, which likely involves simultaneous bidirectional and feedback effects, but are presented here to highlight some key alternative causal relationships consistent with observed PRS trait associations.

## Discussion

This study exploited GWAS data from the PGC and genotype-phenotype data from the UK Biobank, to examine the associations between the common genetic liability for schizophrenia, estimated by PRSs, and a range of behavioural traits. A key feature of our study design is that the UK Biobank sample size allowed us to contrast PRS vs behaviour trends in the unaffected population with the same behaviours in 599 individuals with schizophrenia, in the same cohort. With PRS an incomplete measure of genetic liability, we investigated whether the associations between increasing polygenic risk for schizophrenia and behavioural traits in the undiagnosed general population are predictive of the trait values in medicated and non-medicated schizophrenia cases, who we expect to have higher genetic liability on average.

Almost one-fifth of the associations, which were observed in the general non-diagnosed population sample beyond the typical age of schizophrenia diagnosis (>38 years), were significant at a stringent threshold. Most of these show that increased genetic risk of schizophrenia is associated with negative trait outcomes, such as lower friendship and family satisfaction and greater feelings of guilt and anxiety, reflecting known social cognition challenges in diagnosed and high-risk individuals^31,32^. Thus, the broad findings may support the notion that common genetic risk for schizophrenia manifests as a continuum across the population, with symptoms common to diagnosed schizophrenia cases observed in high-risk unaffected individuals to a greater extent than low-risk unaffected individuals.

While there is justified concern about generating false-positive associations when exploiting polygenic scores that include a large number of null variants, we observed high consistency between the results of best-fit PRS and PRS-based only on genome-wide significant SNPs (Fig. 1), with best-fit PRS having markedly higher explanatory power. Atypical differences between the results of the two types of PRS could highlight potentially confounded associations, as in the case of tea drinking. However, a significant best-fit PRS and non-significant GW-significant PRS may also reflect a peripheral but causal effect, whereby a trait that is only weakly causal of schizophrenia is influenced by variants that are correspondingly of very small effect and thus not among the GW-significant SNPs. Thus, as well as increasing explanatory power, the results from best-fit PRS could provide aetiological insights if evaluated carefully.

The unprecedented size of the UK Biobank as a deeply genotyped-phenotyped data set allowed us to perform a more comprehensive type of analytical comparison. The data were of sufficient size to both conduct a well-powered investigation of PRS–trait relationships at high resolution and to contrast those relationships with corresponding trait values of a sufficient number of individuals with schizophrenia (Fig. 2). While the number of individuals with schizophrenia in the UK Biobank is relatively small in the context of the entire cohort, these data provide an unusual opportunity to perform a comparison of the potential effects of schizophrenia risk between the sub-clinical general population and diagnosed individuals, both those medicated and non-medicated, in the same study population. The trends of association observed in the unaffected and affected sub-cohorts for a fraction of the traits demonstrated that all the results cannot be merely a consequence of disorder misclassification or pre-clinical effects, although these effects are likely to contribute to the observed associations. We hope that our analyses will motivate the collection of samples with sufficiently large numbers of both unaffected and affected individuals, consistently assayed and ideally prospectively collected, so that effects across the disorder liability spectrum can be further explored. Highlighting the downstream effects of schizophrenia diagnosis compared to genetic risk in analyses such as these, which include no cryptic assumptions, should make them a useful counterpart to approaches for causal inference, such as Mendelian Randomisation^9,30^.

However, while there is information to be gleaned from such cross-trait PRS analyses, the results must be interpreted carefully. The schizophrenia PRS based on the PGC schizophrenia GWAS only explains ~ 7% of the variance in schizophrenia liability and overlaps with genetic risk for other psychiatric disorders, such as bipolar^15,33^. The PRS–trait associations reported here are therefore based on small and non-specific effects. The trait variance explained by the schizophrenia PRS has a maximum of only 0.04% (Supplementary Table 2) and for most traits tested is much lower, which means that (1) at best we have some initial insights into the role of behaviour in the genetic risk for schizophrenia and (2) the generation of misleading results due to subtle bias or confounding cannot be entirely ruled out. Furthermore, there is a possibility of collider bias influencing some of the associations due to both ascertainment of the UK Biobank cohort and the covariates that we have controlled for. As the UK Biobank subjects differ on average from the general population, if two associated traits both influence selection into the study, a spurious association may be generated^34^. Adjusting for a collider variable, such as covariate that is correlated with both the PRS and the outcome, can have the same effect. For example, the increased association between schizophrenia PRS and left-hand grip strength when education is controlled for (Supplementary Table 4) could be due to the PRS influencing both the left-hand grip strength and education. Controlling for education would therefore induce the increased association.

Although many of the leading associations, and their directions, are consistent with the literature on schizophrenia aetiology, we highlight the need for more thorough investigation before strong interpretation of any specific association. We illustrate four simple associations (Fig. 3) that could plausibly generate any of the observed PRS–trait associations. Focusing on tea drinking as an example may seem farcical, and was in fact the topic of a popular blog post highlighting the potential problems of PRS associations (with tea drinking a *hypothetical* example)^35^, but is intended to clearly demonstrate the fallacy of assuming that highly significant PRS–trait associations only expose factors on a causal path to the outcome. For this reason, a large-scale systematic study of this type must be considered as exploratory, providing only broad aetiological insights and a screen of potentially interesting links between traits and disorder requiring further investigation. Certainly, none of the results from these analyses should be used to guide present clinical practice.

To exemplify the kind of follow-up analyses that can be performed based on the results of such exploratory studies, we further investigated the association of schizophrenia–PRS and self-reported risk-taking by calculating lifetime within-UK migration patterns and combining those with population density data from the UK National Office of Statistics. These analyses indicated that risk-taking genetics may be a sub-component of the genetic aetiology of schizophrenia due to mediation by migration and/or drug-taking^20–23^. However, this should be considered a hypothesis only, and confirmation of this and an assessment of the contribution of such a relationship to overall schizophrenia prevalence requires dedicated follow-up studies. It is interesting to note that gene–environment correlations of this type effectively inflate both pedigree-based and population-based heritability estimates, since they are regarded as a genetic contribution to the phenotype even if the critical causal agent is environmental.

By mining one of the largest datasets with one of the most comprehensive and detailed behavioural phenotype information, we have arguably produced the most systematic interrogation of the link between genetic risk for schizophrenia and behaviour to date. We have generated a catalogue of 529 schizophrenia PRS–behavioural trait associations, 104 exceeding a stringent multiple testing threshold, demonstrating the influence of genetic risk of schizophrenia on behaviour and lifestyle in the general population. While the large-scale nature of the analyses makes them necessarily preliminary and exploratory, we have demonstrated several ways of gaining greater insights than those derived from the primary associations. We hope that these analytical strategies, and the array of PRS–trait associations generated here, will act as a useful starting point for follow-up investigations to further expose the network linking genetics, behavioural traits and schizophrenia, which may eventually provide targets of early intervention to reduce the risk of schizophrenia.

## Methods

### Base and target data

The base (or discovery) data for this study were the genome-wide association study (GWAS) results from the PGC for schizophrenia^15^. These schizophrenia GWAS results are derived from a sample of up to 36,989 cases and 113,075 controls of European and East Asian ancestry, which yielded 108 independent loci harbouring genome-wide significant associations.

In the present study we analysed target data on up to 307,823 participants (52% females), aged 38–73 years (mean = 56.85, S.D. = 8.06), involved in the UK Biobank baseline assessment (http://www.ukbiobank.ac.uk)^36,37^ and Mental Health Questionnaire. UK Biobank is a health resource for researchers that aims to improve the prevention, diagnosis and treatment of a range of illnesses. The recruitment process was coordinated around 22 centres in the UK (between 2007 and 2010)^37^. Individuals within travelling distance of these centres were identified using NHS patient registers (response rate = 5.47%)^38^. Invitations were sent using a stratified approach to ensure demographic parameters were in concordance with the general population. All participants provided written informed consent and the current study was ethically approved by the UK Biobank Ethics and Governance Council (REC reference 11/NW/0382; UK Biobank application reference 18177).

The traits analysed in the target data were ‘behavioural traits’, liberally defined as any trait that has a substantial behavioural component and included traits across personality, mood, nutrition, physical activity and psychological feelings. Altogether 529 such behavioural traits were analysed (see Supplementary Table 1 for full list), although many of these traits were closely related to each other and thus there is a strong correlation structure among the traits. Details on the 104 significantly associated traits are contained in Supplementary Table 2.

### Base and target data: Quality control and exclusions

Blood samples from 488,366 UK Biobank participants were genotyped using the UK BiLEVE array or the UK Biobank axiom array. Further details on the genotyping and quality control (QC) can be found on the UK Biobank website (http://www.ukbiobank.ac.uk/scientists-3/genetic-data/). In the current study, SNPs were removed if they had missingness > 0.02 and MAF < 0.01. Samples were removed from the dataset if they had missingness > 0.01. We used a subset of European ancestry inferred individuals, defined using 4-means clustering applied to the first two PCs of the genotype data, because PRS are typically underpowered when applied to target samples of a different global ancestry to that of the base data using current PRS methods^5^. Confirming this to be the case here, we repeated the analysis of the top 20 PRS–trait associations (Fig. 1) in the full cohort including 20,798 non-European ancestry inferred individuals and observed less significant PRS–trait associations in 12 of the 20 results despite the substantial increase in sample size (Supplementary Fig. 10). One of each pair of related individuals were removed using a relatedness criterion *KING* coefficient < 0.088. Exclusions based on heterozygosity and missingness were implemented according to UK Biobank recommendations (http://biobank.ctsu.ox.ac.uk/showcase/label.cgi?id=100314). Samples were removed if they were discordant for sex. SNPs deviating from Hardy-Weinberg equilibrium (HWE) were removed at a threshold of *P* < 10^−8^. This QC process resulted in a data set of 560,173 SNPs and 386,192 samples available for analysis.

For our polygenic risk analyses of the unaffected general population, we removed all individuals with an ICD-10 diagnosis of major depressive disorder, bipolar disorder, schizophrenia, and all individuals on antipsychotic medication. There were 307,823 individuals remaining for the analyses of schizophrenia PRS in the unaffected sub-cohort. The analyses that performed comparisons between the unaffected sample and individuals diagnosed with schizophrenia exploited data on 599 individuals in the full UK Biobank data with an ICD-10 hospital diagnosis of ‘schizophrenia, unspecified’ and their antipsychotic medication status. Of these individuals, 297 reported antipsychotic treatment during the verbal interview.

UK Biobank data are available through a procedure described at http://www.ukbiobank.ac.uk/using-the-resource/.

### Primary polygenic risk score analyses

Summary statistics from the schizophrenia GWAS were downloaded from the PGC website (https://www.med.unc.edu/pgc/results-and-downloads). PRSs were generated using *PRSice-2* software (www.prsice.info)^14^. *PRSice* calculates individual risk scores by calculating the sum of disease-associated alleles, weighted by the log odds ratio estimated in the discovery GWAS. SNPs were clumped to minimise their linkage disequilibrium (LD) using an *r*^2^ ≥ 0.1 threshold in sliding windows of 250 kb. PRS on schizophrenia were generated for individuals in the UK Biobank cohort based on the PGC discovery GWAS summary statistics, and then used to predict target phenotypes recorded the UK Biobank. We selected SNPs to calculate the polygenic scores at a *P*-value threshold of *P* < 0.05 based on the PGC’s analysis showing these explained the most case–control variance in 40 leave-one-out analyses^13^. Under a Bonferroni correction for multiple testing that conservatively assumes independence between PRS thresholds and among the base and target traits, we use a significance threshold of 0.05 / (529) = 9.4 × 10^−5^ for the most predictive PRS in our primary analyses, based on the 529 target traits tested. For further stringency, given the risk of detecting significant associations that are a result of subtle confounding in such large sample sizes, we only declare significance at *P* < 1 × 10^−7^. Details of the 104 phenotypes that show an association with the most predictive PRS at *P* < 1 × 10^−7^ in the target data are provided in Supplementary Table 2. Associations were examined in regression models for the binary (logistic) and quantitative (linear) target traits, adjusting for age, sex, Townsend deprivation index and the first 15 PCs of the genotype data to control for population stratification, with additional adjustments described in the text for relevant analyses. The PCs were provided as part of the UK Biobank genotype data release. As further reassurance that the observed associations are genuine, we repeated the analyses using PRS based only on the 108 sentinel genome-wide (GW) significant variants for schizophrenia.

### Quantile plots

Quantile plots were generated by creating 20 bins of the screened subjects based on their PRSs, with the lowest quantile containing the 5% of the subjects with the lowest genetic liability up to the 5% with the highest. Each trait phenotype was then regressed on the 20 quantiles using linear and logistic regressions on continuous and binary traits, respectively, with the 11^th^ quantile used as the reference. In all, 15 PCs, age, sex, batch and centre were included as covariates in each regression. For the schizophrenia cases, a 21^st^ quantile was used in the regressions and in the medicated/non-medicated plots a further two were used.

### Migration and substance abuse analyses

The UK Biobank data included birth and current residence (at baseline) location variables, under our application, defined using the British National Grid referencing system, corresponding to northerly and easterly positions, with a reference point close to the Isles of Scilly. We used these locations to characterise migration patterns of UK Biobank individuals within the UK. First, the Euclidean distance travelled between birth and current residence was calculated using the northerly/easterly co-ordinates of each; the distance is calculated to the nearest kilometre to prevent identification (mean distance travelled = 84.9 km, median = 15.0 km). Next, population density measures were derived by matching the co-ordinates of each participant to population density information held on national databases relating to each local authority district. Population density statistics and boundary data for local authority districts were downloaded from the office of national statistics (https://www.ons.gov.uk), based on the 2011 UK Census data. The R packages sp and rgdal were then used to retrieve the appropriate population density metrics for each participant according to their location co-ordinates. Thus, the population density measures for birth place (mean = 2376 per km^2^, median = 1897 per km^2^), current residence (mean = 2059 per km^2^, median = 1415 per km^2^) and their difference (mean = −316.7 per km^2^, median = 0), were derived for each participant and, again, corresponded to the local postcode area. Time spent at current residence was available directly as a UK Biobank variable (mean = 17.8 years, median = 16 years). Substance abuse, defined as those with an ICD-10 hospital diagnosis or self-reported substance abuse (total UK Biobank sample: cases = 4033, controls = 498,631), was also available.

Self-reported risk-taking was tested for association with the migration phenotypes using linear regression and with substance abuse using logistic regression. The control phenotypes that were used in this follow-up analyses, also tested via linear and logistic regressions, were: breast fed (binary: cases = 277,671, controls = 106,156); birth weight known (binary: cases = 277,076, controls = 224,726); birth weight (mean = 3.12 kg, median = 3.32 kg); left hand grip (mean = 29.55, median = 28); leg pain on walking (cases = 39,759, control = 130,710); blood pressure device ID and month attended assessment centre. Each of these phenotypes were then tested for an association with the schizophrenia PRS. All of the analyses were adjusted for age, sex, Townsend deprivation index and educational attainment, as described in the main text and corresponding table captions.

### Reporting summary

Further information on experimental design is available in the Nature Research Reporting Summary linked to this paper.

## Supplementary information

Supplementary material.

Reporting summary.

## Data Availability

Schizophrenia GWAS summary statistics are publicly available from the PGC (https://www.med.unc.edu/pgc/download-results/). The data from UK Biobank application reference 18177 were provided under license by UK Biobank, who is the owner of the data. The individual-level genotype and phenotype data used in this study can be provided by the UK Biobank (http://www.ukbiobank.ac.uk/) pending scientific review and a completed material transfer agreement (requests for these data should be submitted to the UK Biobank).

## Code availability

Software code for *PRSice-2* is available at https://www.prsice.info/. All other code used is available upon request.
