# Supplementary material for: Investigating the effects of genetic risk of schizophrenia on behavioural traits
Source: NPJ Schizophr. 2021 Jan 22;7:2. doi: 10.1038/s41537-020-00131-2 (PMC7822841; doi:10.1038/s41537-020-00131-2)
Supplement: Supplementary file 1 — Supplementary material. [file 41537_2020_131_MOESM1_ESM.pdf]

Supplementary Material

Supplementary Table 1. All 529 behavioural traits used in the analysis, as described by the UK Biobank for completeness.

Ever thought that life not worth living
Self harmed
Self harmed in past year
Number of times self harmed
Attempted suicide
Attempted suicide in past year
Ever contemplated self harm
Contemplated self harm in past year
Felt hated by family member as a child
Physically abused by family as a child
Sexually molested as a child
Belittlement by partner or ex partner as an adult
Physical violence by partner or ex partner as an adult
Sexual interference by partner or ex partner without consent as an adult
Carer support indicators
Detention categories
Legal statuses
Mental categories
History of psychiatric care on admission
Number of correct matches in round 0 1
Number of correct matches in round 0 2
Number of correct matches in round 0 3
Number of incorrect matches in round 0 1
Number of incorrect matches in round 0 2
Number of incorrect matches in round 0 3
Time to complete round 0 1
Time to complete round 0 2
Time to complete round 0 3
Own or rent accommodation lived in
Length of time at current address
Number in household
Number of vehicles in household
Average total household income before tax
Time employed in main current job
Length of working week for main job
Frequency of travelling from home to job workplace
Distance between home and job workplace
Job involves mainly walking or standing
Job involves heavy manual or physical work
Job involves shift work
Age completed full time education
Number of days week walked 10 minutes
Duration of walks
Number of days week of moderate physical activity 10 minutes
Duration of moderate activity
Number of days week of vigorous physical activity 10 minutes
Duration of vigorous activity
Usual walking pace
Frequency of stair climbing in last 4 weeks
Frequency of walking for pleasure in last 4 weeks
Duration walking for pleasure
Frequency of strenuous sports in last 4 weeks
Duration of strenuous sports
Frequency of light DIY in last 4 weeks
Duration of light DIY
Frequency of friend family visits

**Time spend outdoors in summer**
**Time spent outdoors in winter**
**Time spent watching television TV**
**Time spent using computer**
**Time spent driving**
**Drive faster than motorway speed limit**
**Length of mobile phone use**
**Weekly usage of mobile phone in last 3 months**
**Hands free device speakerphone use with mobile phone in last 3 month**
**Difference in mobile phone use compared to two years previously**
**Usual side of head for mobile phone use**
**Sleep duration**
**Getting up in morning**
**Morning evening person chronotype**
**Nap during day**
**Sleeplessness insomnia**
**Snoring**
**Daytime dozing sleeping narcolepsy**
**Current tobacco smoking**
**Past tobacco smoking**
**Smoking smokers in household**
**Exposure to tobacco smoke at home**
**Exposure to tobacco smoke outside home**
**Cooked vegetable intake**
**Salad raw vegetable intake**
**Fresh fruit intake**
**Dried fruit intake**
**Oily fish intake**
**Non oily fish intake**
**Processed meat intake**
**Poultry intake**
**Beef intake**
**Lamb mutton intake**
**Pork intake**
**Cheese intake**
**Milk type used**
**Salt added to food**
**Tea intake**
**Coffee intake**
**Water intake**
**Major dietary changes in the last 5 years**
**Variation in diet**
**Alcohol intake frequency**
**Average weekly red wine intake**
**Average weekly beer plus cider intake**
**Average weekly spirits intake**
**Average weekly fortified wine intake**
**Alcohol intake versus 10 years previously**
**Country of birth UK elsewhere**
**Breastfed as a baby**
**Skin colour**
**Adopted as a child**
**Maternal smoking around birth**
**Father still alive**
**Mother still alive**
**Mood swings**
**Miserableness**
**Irritability**
**Sensitivity hurt feelings**
**Fed up feelings**
**Nervous feelings**
**Worrier anxious feelings**
**Tense 'highly strung'**

**Worry too long after embarrassment**
**Suffer from 'nerves'**
**Loneliness isolation**
**Guilty feelings**
**Risk taking**
**Frequency of depressed mood in last 2 weeks**
**Frequency of unenthusiasm disinterest in last 2 weeks**
**Frequency of tenseness restlessness in last 2 weeks**
**Frequency of tiredness lethargy in last 2 weeks**
**Seen doctor GP for nerves anxiety tension or depression**
**Seen a psychiatrist for nerves anxiety tension or depression**
**Able to confide**
**Answered sexual history questions**
**Age first had sexual intercourse**
**Lifetime number of sexual partners**
**Ever had same sex intercourse**
**Overall health rating**
**Plays computer games**
**Use of sun uv protection**
**Frequency of solarium sunlamp use**
**Falls in the last year**
**Number of children fathered**
**Frequency of heavy DIY in last 4 weeks**
**Duration of heavy DIY**
**Light smokers at least 100 smokes in lifetime**
**Reason for reducing amount of alcohol drunk**
**Number of live births**
**Ever had stillbirth spontaneous miscarriage or termination**
**Ever taken oral contraceptive pill**
**Age started oral contraceptive pill**
**Ever used hormone replacement therapy HRT**
**Age started smoking in former smokers**
**Type of tobacco previously smoked**
**Number of cigarettes previously smoked daily**
**Age stopped smoking**
**Ever stopped smoking for 6 months**
**Number of unsuccessful stop smoking attempts**
**Likelihood of resuming smoking**
**Caffeine drink within last hour**
**Pregnant**
**Smoked cigarette or pipe within last hour**
**Job involve night shift work**
**Age started smoking in current smokers**
**Type of tobacco currently smoked**
**Number of cigarettes currently smoked daily current cigarette smokers**
**Time from waking to first cigarette**
**Difficulty not smoking for 1 day**
**Ever tried to stop smoking**
**Wants to stop smoking**
**Smoking compared to 10 years previous**
**Frequency of other exercises in last 4 weeks**
**Duration of other exercises**
**Year immigrated to UK United Kingdom**
**Lifetime number of same sex sexual partners**
**Age when last ate meat**
**Former alcohol drinker**
**Number of pregnancy terminations**
**Reason former drinker stopped drinking alcohol**
**Completion status of numeric memory test**
**Maximum digits remembered correctly**
**Number of rounds of numeric memory test performed**
**Average monthly red wine intake**
**Average monthly champagne plus white wine intake**

**Average monthly beer plus cider intake**
**Average monthly spirits intake**
**Average monthly fortified wine intake**
**Average monthly intake of other alcoholic drinks**
**Happiness**
**Work job satisfaction**
**Health satisfaction**
**Family relationship satisfaction**
**Friendships satisfaction**
**Financial situation satisfaction**
**Ever depressed for a whole week**
**Longest period of depression**
**Number of depression episodes**
**Ever unenthusiastic disinterested for a whole week**
**Ever manic hyper for 2 days**
**Ever highly irritable argumentative for 2 days**
**Private healthcare**
**Noisy workplace**
**Loud music exposure frequency**
**Attempted fluid intelligence FI test**
**FI1 numeric addition test**
**FI2 identify largest number**
**FI3 word interpolation**
**FI4 positional arithmetic**
**FI5 family relationship calculation**
**FI6 conditional arithmetic**
**FI7 synonym**
**FI8 chained arithmetic**
**Average weekly intake of other alcoholic drinks**
**Longest period of unenthusiasm disinterest**
**Number of unenthusiastic disinterested episodes**
**Length of longest manic irritable episode**
**Severity of manic irritable episodes**
**FI10 arithmetic sequence recognition**
**FI11 antonym**
**FI12 square sequence recognition**
**FI13 subset inclusion logic**
**Previously smoked cigarettes on most all days**
**Able to walk or cycle unaided for 10 minutes**
**Number of cigarettes previously smoked daily current cigar pipe smokers**
**Age stopped smoking cigarettes current cigar pipe or previous cigarette smoker**
**Email access**
**Fluid intelligence score**
**Prospective memory result**
**Mean time to correctly identify matches**
**Job code deduced**
**Smoking status**
**Alcohol drinker status**
**Home area population density urban or rural**
**Current employment status corrected**
**Cascot confidence score**
**Bipolar disorder status**
**Single episode of probable major depression**
**Probable recurrent major depression moderate**
**Probable recurrent major depression severe**
**Bipolar and major depression status**
**Neuroticism score**
**Number of fluid intelligence questions attempted within time limit**
**Number of correct matches in round**
**Number of correct matches in round 0 1**
**Number of correct matches in round 0 2**
**Number of incorrect matches in round**
**Number of incorrect matches in round 0 1**

**Number of incorrect matches in round 0 2**
**Time to complete round**
**Time to complete round 0 1**
**Time to complete round 0 2**
**When pairs test completed**
**When fluid intelligence test completed**
**When trail making test completed**
**When symbol digit substitution test completed**
**When numeric memory test completed**
**When initial questions completed**
**Ever smoked**
**FI1 numeric addition test**
**FI2 identify largest number**
**FI3 word interpolation**
**FI4 positional arithmetic**
**FI5 family relationship calculation**
**FI6 conditional arithmetic**
**FI7 synonym**
**FI8 chained arithmetic**
**FI9 concept interpolation**
**FI10 arithmetic sequence recognition**
**FI11 antonym**
**FI12 square sequence recognition**
**FI13 subset inclusion logic**
**Fluid intelligence score**
**Number of fluid intelligence questions attempted within time limit**
**FI14 alphanumeric substitution**
**Number of symbol digit matches attempted**
**Maximum digits remembered correctly**
**Tobacco smoking**
**Age of stopping smoking**
**Amount of tobacco currently smoked**
**Number of jobs held**
**Nitrogen dioxide air pollution 2010**
**Nitrogen oxides air pollution 2010**
**Particulate matter air pollution pm10 2010**
**Particulate matter air pollution pm2 5 2010**
**Particulate matter air pollution pm2 5 absorbance 2010**
**Particulate matter air pollution 2 5 10um 2010**
**Traffic intensity on the nearest road**
**Inverse distance to the nearest road**
**Traffic intensity on the nearest major road**
**Inverse distance to the nearest major road**
**Total traffic load on major roads**
**Close to major road**
**Sum of road length of major roads within 100m**
**Nitrogen dioxide air pollution 2005**
**Nitrogen dioxide air pollution 2006**
**Nitrogen dioxide air pollution 2007**
**Particulate matter air pollution pm10 2007**
**Average daytime sound level of noise pollution**
**Average evening sound level of noise pollution**
**Average night time sound level of noise pollution**
**Average 16 hour sound level of noise pollution**
**Average 24 hour sound level of noise pollution**
**Drinking water intake**
**Alcohol consumed**
**Time spent doing vigorous physical activity**
**Time spent doing moderate physical activity**
**Time spent doing light physical activity**
**Ever addicted to any substance or behaviour**
**Amount of alcohol drunk on a typical drinking day**
**Ever physically dependent on alcohol**

Ever had known person concerned about or recommend reduction of alcohol consumption
Ever addicted to alcohol
Frequency of failure to fulfil normal expectations due to drinking alcohol in last year
Frequency of memory loss due to drinking alcohol in last year
Frequency of feeling guilt or remorse after drinking alcohol in last year
Age when known person last commented about drinking habits
Ever been injured or injured someone else through drinking alcohol
Frequency of needing morning drink of alcohol after heavy drinking session in last year
Frequency of inability to cease drinking in last year
Frequency of drinking alcohol
Ongoing addiction to alcohol
Frequency of consuming six or more units of alcohol
Tense sore or aching muscles during worst period of anxiety
Impact on normal roles during worst period of anxiety
Difficulty concentrating during worst period of anxiety
Longest period spent worried or anxious
Ever felt worried tense or anxious for most of a month or longer
More irritable than usual during worst period of anxiety
Keyed up or on edge during worst period of anxiety
Ever worried more than most people would in similar situation
Restless during period of worst anxiety
Frequent trouble falling or staying asleep during worst period of anxiety
Professional informed about anxiety
Easily tired during worst period of anxiety
Ever addicted to a behaviour or miscellanous
Ongoing behavioural or miscellanous addiction
Age at first episode of depression
Age at last episode of depression
Difficulty concentrating during worst depression
Fraction of day affected during worst episode of depression
Thoughts of death during worst depression
Duration of worst depression
Frequency of depressed days during worst episode of depression
Impact on normal roles during worst period of depression
Ever had prolonged loss of interest in normal activities
Lifetime number of depressed periods
Depression possibly related to childbirth
Ever had prolonged feelings of sadness or depression
Depression possibly related to stressful or traumatic event
Professional informed about depression
Feelings of tiredness during worst episode of depression
Feelings of worthlessness during worst period of depression
Ever taken cannabis
Maximum frequency of taking cannabis
Age when last took cannabis
Ever addicted to illicit or recreational drugs
Illicit or recreational drugs addiction or dependence ongoing
General happiness
General happiness with own health
Belief that own life is meaningful
Age when first had unusual or psychotic experience
Distress caused by unusual or psychotic experiences
Ever heard an unreal voice
Number of times heard an unreal voice
Ever prescribed a medication for unusual or psychotic experiences
Frequency of unusual or psychotic experiences in past year
Ever believed in an unreal conspiracy against self
Number of times believed in an unreal conspiracy against self
Ever seen an unreal vision
Number of times seen an unreal vision
Ever believed in unreal communications or signs
Number of times believed in unreal communications or signs
Ever talked to a health professional about unusual or psychotic experiences

**Felt loved as a child**
**Someone to take to doctor when needed as a child**
**Longest period of mania or irritability**
**Severity of problems due to mania or irritability**
**Felt irritable or had angry outbursts in past month**
**Avoided activities or situations because of previous stressful experience in past month**
**Felt distant from other people in past month**
**Repeated disturbing thoughts of stressful experience in past month**
**Felt very upset when reminded of stressful experience in past month**
**Ever sought or received professional help for mental distress**
**Ever suffered mental distress preventing usual activities**
**Ever had period of mania excitability**
**Ever had period extreme irritability**
**Ever addicted to prescription or over the counter medication**
**Prescription or over the counter medication addiction or dependence ongoing**
**Recent easy annoyance or irritability**
**Recent feelings of nervousness or anxiety**
**Recent feelings of inadequacy**
**Recent trouble concentrating on things**
**Recent inability to stop or control worrying**
**Recent feelings of depression**
**Recent poor appetite or overeating**
**Recent feelings of foreboding**
**Recent thoughts of suicide or self harm**
**Recent lack of interest or pleasure in doing things**
**Recent trouble relaxing**
**Recent restlessness**
**Trouble falling or staying asleep or sleeping too much**
**Recent changes in speed amount of moving or speaking**
**Recent feelings of tiredness or low energy**
**Recent worrying too much about different things**
**Been in a confiding relationship as an adult**
**Able to pay rent mortgage as an adult**
**Been in serious accident believed to be life threatening**
**Been involved in combat or exposed to war zone**
**Diagnosed with life threatening illness**
**Victim of physically violent crime**
**Witnessed sudden violent death**
**Did your sleep change**
**Trouble falling asleep**
**Sleeping too much**
**Waking too early**
**Weight change during worst episode of depression**
**Frequency of difficulty controlling worry during worst period of anxiety**
**Worried most days during period of worst anxiety**
**Frequency of inability to stop worrying during worst period of anxiety**
**Multiple worries during worst period of anxiety**
**Difficulty stopping worrying during worst period of anxiety**
**Stronger worrying than other people during period of worst anxiety**
**Number of things worried about during worst period of anxiety**
**Current employment status employed**
**Current employment status retired**
**Current employment status family**
**Current employment status disabled**
**Current employment status unemployed**
**Current employment status voluntary**
**Current employment status student**
**Heating types in home gas**
**Heating types in home electricity**
**Heating types in home oil**
**Heating types in home paraffin**
**Heating types in home solid fuel**
**Heating types in home open fire**

How are people in household related to participant partner
How are people in household related to participant children
How are people in household related to participant siblings
How are people in household related to participant parents
How are people in household related to participant grandparents
How are people in household related to participant grandchildren
How are people in household related to participant other related
How are people in household related to participant other unrelated
Illness injury bereavement stress in last 2 years illness self
Illness injury bereavement stress in last 2 years illness relative
Illness injury bereavement stress in last 2 years death relative
Illness injury bereavement stress in last 2 years death spouse
Illness injury bereavement stress in last 2 years divorce
Illness injury bereavement stress in last 2 years financial difficulties
Leisure social activities sports
Leisure social activities pub
Leisure social activities religion
Leisure social activities adult class
Leisure social activities other
Manic hyper symptoms more active
Manic hyper symptoms more talkative
Manic hyper symptoms less sleep
Manic hyper symptoms more creative
Manic hyper symptoms all
Mineral and other dietary supplements fish oil
Mineral and other dietary supplements glucosamine
Mineral and other dietary supplements calcium
Mineral and other dietary supplements zinc
Mineral and other dietary supplements iron
Mineral and other dietary supplements selenium
Never eat eggs dairy wheat sugar eggs
Never eat eggs dairy wheat sugar dairy
Never eat eggs dairy wheat sugar wheat
Never eat eggs dairy wheat sugar sugar
Never eat eggs dairy wheat sugar none
Qualifications university
Qualifications A levels
Qualifications GCSEs
Qualifications CSEs
Qualifications NVQ
Qualifications other
Transport type for commuting to job workplace car
Transport type for commuting to job workplace walk
Transport type for commuting to job workplace public transport
Transport type for commuting to job workplace cycle
Type of special diet followed gluten free
Type of special diet followed lactose free
Type of special diet followed low calorie
Type of special diet followed vegetarian
Type of special diet followed vegan
Type of special diet followed other
Types of physical activity in last 4 weeks walking
Types of physical activity in last 4 weeks other
Types of physical activity in last 4 weeks light DIY
Types of physical activity in last 4 weeks heavy DIY
Types of transport used excluding work car
Types of transport used excluding work walk
Types of transport used excluding work public transport
Types of transport used excluding work cycle
Vitamin and mineral supplements A
Vitamin and mineral supplements B
Vitamin and mineral supplements C
Vitamin and mineral supplements D

**Vitamin and mineral supplements E**
**Vitamin and mineral supplements folic acid**
**Vitamin and mineral supplements multi**
**Vitamin and or mineral supplement use_dichotomised multi**
**Vitamin and or mineral supplement use_dichotomised multi iron**
**Vitamin and or mineral supplement use_dichotomised multi calcium**
**Vitamin and or mineral supplement use_dichotomised multi minerals**
**Vitamin and or mineral supplement use_dichotomised fish oil**
**Vitamin and or mineral supplement use_dichotomised glucosamine**
**Vitamin and or mineral supplement use_dichotomised evening primrose**
**Vitamin and or mineral supplement use_dichotomised A**
**Vitamin and or mineral supplement use_dichotomised B6**
**Vitamin and or mineral supplement use_dichotomised B12**
**Vitamin and or mineral supplement use_dichotomised C**
**Vitamin and or mineral supplement use_dichotomised D**
**Vitamin and or mineral supplement use_dichotomised E**
**Vitamin and or mineral supplement use_dichotomised folic acid**
**Vitamin and or mineral supplement use_dichotomised chromium**
**Vitamin and or mineral supplement use_dichotomised magnesium**
**Vitamin and or mineral supplement use_dichotomised selenium**
**Vitamin and or mineral supplement use_dichotomised calcium**
**Vitamin and or mineral supplement use_dichotomised iron**
**Vitamin and or mineral supplement use_dichotomised zinc**
**Vitamin and or mineral supplement use_dichotomised other**
**Why reduced smoking illness**
**Why reduced smoking doctor**
**Why reduced smoking health**
**Why reduced smoking financial**
**Why stopped smoking illness**
**Why stopped smoking doctor**
**Why stopped smoking health**
**Why stopped smoking financial**

Supplementary Table 2: The 104 target traits associated with schizophrenia polygenic risk scores (PRSs) at $P < 1 \times 10^{-7}$ in the unaffected sub-cohort of the UK Biobank.

Trait	Direction of effect	P_t<0.05 PRS R2	P_t<0.05 PRS *P*-value	GW-sig *P*-value	*N*	Unaffected vs. cases different *P*-value	Trait type
**Number of incorrect matches in round 2 (*Pair matching errors*)**	**+**	**0.001**	**2.94×10^{-82}**	**3.14×10^{-13}**	**307395**	**7.20×10^{-01}**	**Cognitive**
Number of fluid intelligence questions attempted within time limit	-	0.003	2.32×10^{-76}	1.11×10^{-27}	99466	9.37×10^{-02}	Cognitive
Seen doctor GP for nerves anxiety tension or depression	+	0.002	9.99×10^{-75}	1.57×10^{-15}	305607	2.07×10^{-176}	Affective
Plays computer games	-	0.001	2.71×10^{-59}	2.56×10^{-08}	307479	4.60×10^{-06}	Behavioural
Seen a psychiatrist for nerves anxiety tension or depression	+	0.002	1.59×10^{-58}	1.24×10^{-16}	306579	1.15×10^{-255}	Affective
Time to complete round 2	+	0.001	2.39×10^{-57}	1.15×10^{-16}	307395	2.46×10^{-02}	Cognitive
Mean time to correctly identify matches	+	0.001	2.36×10^{-54}	1.70×10^{-07}	305797	1.98×10^{-25}	Cognitive
**Oily fish intake**	**+**	**0.001**	**7.22×10^{-54}**	**3.62×10^{-20}**	**306254**	**5.01×10^{-04}**	**Behavioural**
**Fluid intelligence score[+]**	**-**	**0.002**	**2.18×10^{-53}**	**3.43×10^{-15}**	**99466**	**1.56×10^{-17}**	**Cognitive**
**Nervous feelings**	**+**	**0.001**	**1.25×10^{-46}**	**2.09×10^{-17}**	**300070**	**1.87×10^{-67}**	**Affective**
**Getting up in morning**	**-**	**0.001**	**5.05×10^{-45}**	**2.26×10^{-05}**	**307056**	**3.12×10^{-37}**	**Disorganisation**
**Ever depressed for a whole week**	**+**	**0.003**	**2.58×10^{-44}**	**8.41×10^{-11}**	**99725**	**3.90×10^{-30}**	**Affective**
Time to complete round 1	+	0.001	3.36×10^{-44}	8.79×10^{-11}	307395	2.66×10^{-07}	Cognitive
Ever unenthusiastic disinterested for a whole week	+	0.003	1.07×10^{-43}	8.00×10^{-10}	97754	7.98×10^{-28}	Affective
Frequency of tenseness restlessness in last 2 weeks	+	0.001	1.67×10^{-43}	2.02×10^{-09}	296554	2.51×10^{-38}	Affective
**BMI**	**-**	**0.001**	**1.05×10^{-41}**	**2.82×10^{-13}**	**306919**	**4.93×10^{-18}**	**Disorganisation/behaviour**
Number of incorrect matches in round 1	+	0.001	6.88×10^{-40}	1.16×10^{-07}	307395	2.28×10^{-06}	Cognitive
**Neuroticism score (*Neuroticism*)**	**+**	**0.001**	**7.82×10^{-40}**	**2.16×10^{-12}**	**250454**	**5.05×10^{-64}**	**Affective**
**Number of days week of moderate physical activity 10 minutes (*Moderate physical activity*)**	**+**	**0.001**	**2.03×10^{-38}**	**3.85×10^{-14}**	**293844**	**2.15×10^{-09}**	**Behavioural**
**Self-reported risk taking (*Risk taking)***	**+**	**0.001**	**3.00×10^{-38}**	**5.78×10^{-07}**	**297101**	**1×10^{-308}**	**Behavioural**

Reason for reducing amount of alcohol drunk	-	0.001	$2.46x10^{-35}$	$4.28x10^{-03}$	112644	$5.60x10^{-23}$	Behavioural
**Family relationship satisfaction**	**+**	**0.001**	**$6.65x10^{-35}$**	**$1.92x10^{-06}$**	**100952**	**$1.26x10^{-22}$**	**Affective**
**Current smoking status (*Smoking*)**	**+**	**0.000**	**$5.45x10^{-34}$**	**$6.04x10^{-03}$**	**306505**	**$5.02x10^{-30}$**	**Behavioural**
Why stopped smoking health	+	0.001	$9.60x10^{-34}$	$9.55x10^{-13}$	59132	$3.28x10^{-03}$	Behavioural
Worrier anxious feelings	+	0.001	$1.70x10^{-33}$	$2.53x10^{-13}$	299507	$2.68x10^{-11}$	Affective
Time to answer	+	0.001	$3.79x10^{-31}$	$6.69x10^{-09}$	101682	$7.45x10^{-02}$	Cognitive
**Guilty feelings**	**+**	**0.001**	**$5.47x10^{-30}$**	**$2.22x10^{-09}$**	**299852**	**$5.33x10^{-25}$**	**Affective**
Frequency of depressed mood in last 2 weeks	+	0.000	$1.27x10^{-29}$	$7.21x10^{-10}$	295142	$1.68x10^{-40}$	Affective
Frequency of unenthusiasm disinterest in last 2 weeks	+	0.000	$1.06x10^{-28}$	$3.10x10^{-07}$	298216	$2.94x10^{-38}$	Affective
**Tea intake**	**+**	**0.000**	**$1.69x10^{-28}$**	**$4.23x10^{-01}$**	**297475**	**$1.44x10^{-03}$**	**Behavioural**
**Felt loved as a child**	**-**	**0.001**	**$3.29x10^{-28}$**	**$1.39x10^{-01}$**	**83605**	**$5.37x10^{-04}$**	**Affective**
Past tobacco smoking	-	0.000	$1.01x10^{-26}$	$1.72x10^{-04}$	288806	$2.35x10^{-02}$	Behavioural
Non-oily fish intake	+	0.000	$9.22x10^{-26}$	$2.60x10^{-12}$	306525	$5.62x10^{-07}$	Behavioural
Frequency of tiredness lethargy in last 2 weeks	+	0.000	$1.19x10^{-24}$	$3.78x10^{-06}$	298429	$2.65x10^{-36}$	Motor
**Friendship satisfaction**	**+**	**0.001**	**$2.17x10^{-24}$**	**$5.67x10^{-04}$**	**100886**	**$6.72x10^{-12}$**	**Affective**
**Length of time at current address**	**-**	**0.000**	**$7.52x10^{-24}$**	**$2.61x10^{-06}$**	**300865**	**$5.44x10^{-04}$**	**Behavioural**
Number of correct matches in round 2	-	0.000	$1.26x10^{-22}$	$1.29x10^{-03}$	307395	$7.97x10^{-18}$	Cognitive
**Cooked vegetable intake**	**+**	**0.000**	**$6.35x10^{-22}$**	**$5.21x10^{-04}$**	**298869**	**$7.70x10^{-02}$**	**Behavioural**
**Sensitivity hurt feelings**	**+**	**0.000**	**$1.74x10^{-21}$**	**$8.72x10^{-10}$**	**298692**	**$5.05x10^{-12}$**	**Affective**
Avoided activities or situations because of previous stressful experience in past month	+	0.001	$5.06x10^{-21}$	$7.16x10^{-02}$	83805	$2.47x10^{-05}$	Affective
PM initial answer	-	0.001	$1.04x10^{-20}$	$2.99x10^{-06}$	101664	$3.83x10^{-08}$	Cognitive
Number of correct matches in round 1	-	0.000	$2.38x10^{-20}$	$9.99x10^{-03}$	307396	$5.21x10^{-17}$	Cognitive
Bipolar and major depression status	+	0.001	$2.65x10^{-20}$	$2.33x10^{-07}$	73573	$2.71x10^{-18}$	Affective
Duration screen displayed	+	0.001	$3.47x10^{-20}$	$1.76x10^{-06}$	101682	$8.13x10^{-07}$	Cognitive
Happiness	+	0.001	$5.45x10^{-20}$	$7.57x10^{-06}$	101565	$4.16x10^{-13}$	Affective
Ever felt worried tense or anxious for most of a month or longer	+	0.002	$5.68x10^{-19}$	$2.72x10^{-03}$	80410	$2.96x10^{-06}$	Affective
Vitamin and mineral supplements C	+	0.001	$6.41x10^{-19}$	$3.84x10^{-07}$	307812	$6.25x10^{-01}$	Behavioural
**Nap during day**	**+**	**0.000**	**$6.79x10^{-19}$**	**$6.98x10^{-06}$**	**307469**	**$4.75x10^{-34}$**	**Motor/disorganisation**
How are people in household related	-	0.000	$1.06x10^{-18}$	$2.21x10^{-02}$	307812	$1.60x10^{-106}$	Behavioural

to participant partner							
Repeated disturbing thoughts of stressful experience in past month	+	0.001	$1.08x10^{-18}$	$2.97x10^{-02}$	83808	$4.51x10^{-04}$	Delusions
Time spent watching television TV	-	0.000	$1.62x10^{-18}$	$6.04x10^{-01}$	290951	$2.90x10^{-09}$	Behavioural
Number of days week of vigorous physical activity 1 minutes	+	0.000	$3.94x10^{-18}$	$1.02x10^{-05}$	293799	$3.81x10^{-08}$	Behavioural/motor
Ever suffered mental distress preventing usual activities	+	0.002	$3.92x10^{-17}$	$7.80x10^{-02}$	82792	$1.53x10^{-25}$	Affective
Number of vehicles in household	-	0.000	$5.19x10^{-17}$	$2.11x10^{-02}$	305805	$2.81x10^{-138}$	Behavioural
Pork intake	-	0.000	$8.12x10^{-17}$	$3.52x10^{-03}$	306068	$9.79x10^{-01}$	Behavioural
Types of transport used excluding work car	-	0.000	$1.23x10^{-16}$	$5.11x10^{-02}$	307812	$2.79x10^{-90}$	Behavioural
Felt very upset when reminded of stressful experience in past month	+	0.001	$1.30x10^{-16}$	$4.59x10^{-01}$	83794	$6.14x10^{-05}$	Affective
Age first had sexual intercourse	-	0.000	$5.03x10^{-16}$	$4.67x10^{-04}$	269875	$7.74x10^{-01}$	Behavioural
Sleep duration	+	0.000	$6.99x10^{-16}$	$3.13x10^{-04}$	306190	$1.06x10^{-08}$	Motor
Mineral and other dietary supplements zinc	+	0.001	$1.45x10^{-15}$	$5.55x10^{-05}$	307812	$6.85x10^{-03}$	Behavioural
Qualifications GCSx10s	-	0.000	$3.69x10^{-15}$	$2.58x10^{-01}$	307812	$1.72x10^{-03}$	Behavioural
Vitamin and mineral supplements x10	+	0.001	$7.28x10^{-15}$	$1.46x10^{-04}$	307812	$2.61x10^{-01}$	Behavioural
Vitamin and mineral supplements A	+	0.001	$4.74x10^{-14}$	$1.08x10^{-06}$	307812	$1.69x10^{-02}$	Behavioural
Leisure social activities adult class	+	0.000	$9.18x10^{-14}$	$1.77x10^{-03}$	307812	$1.51x10^{-02}$	Behavioural
Overall health rating	+	0.000	$1.15x10^{-13}$	$2.97x10^{-01}$	306588	$5.15x10^{-68}$	Behavioural
Loneliness isolation	+	0.000	$4.98x10^{-13}$	$1.81x10^{-02}$	303135	$2.40x10^{-48}$	Affective
Worry too long after embarrassment	+	0.000	$1.11x10^{-12}$	$3.46x10^{-02}$	295015	$1.12x10^{-15}$	Affective
Number of pregnancy terminations	+	0.001	$1.76x10^{-12}$	$2.11x10^{-03}$	48343	$7.68x10^{-03}$	Behavioural
Ever had prolonged loss of interest in normal activities	+	0.001	$2.75x10^{-12}$	$1.22x10^{-04}$	83678	$2.25x10^{-16}$	Negative
Number of attempts	+	0.000	$3.19x10^{-12}$	$1.10x10^{-02}$	101682	$9.65x10^{-03}$	Affective
Ever sought or received professional help for mental distress	+	0.001	$3.37x10^{-12}$	$1.00x10^{-02}$	83656	$1x10^{-308}$	Affective

Qualifications university	+	0.000	$4.44x10^{-12}$	$3.36x10^{-01}$	307812	$1.20x10^{-22}$	Behavioural
Loud music exposure frequency	+	0.000	$7.09x10^{-12}$	$1.92x10^{-02}$	100541	$8.46x10^{-05}$	Behavioural
Felt hated by family member as a child	+	0.001	$8.49x10^{-12}$	$2.72x10^{-01}$	83737	$8.98x10^{-04}$	Affective
Length of mobile phone use	-	0.000	$3.45x10^{-11}$	$3.82x10^{-01}$	303951	$1.85x10^{-33}$	Behavioural
Belittlement by partner or ex-partner as an adult	+	0.001	$4.13x10^{-11}$	$3.68x10^{-01}$	83687	$4.03x10^{-03}$	Behavioural
Current tobacco smoking	+	0.000	$6.06x10^{-11}$	$1.67x10^{-01}$	307490	$9.50x10^{-27}$	Behavioural
Coffee intake	-	0.000	$6.88x10^{-11}$	$1.32x10^{-05}$	285407	$1.71x10^{-07}$	Behavioural
Vitamin and mineral supplements B	+	0.000	$1.20x10^{-10}$	$4.85x10^{-03}$	307812	$1.10x10^{-03}$	Behavioural
Recent feelings of nervousness or anxiety	+	0.000	$1.43x10^{-10}$	$3.90x10^{-05}$	83610	$2.30x10^{-03}$	Affective
Ever thought that life not worth living	+	0.000	$1.99x10^{-10}$	$1.80x10^{-01}$	83477	$4.46x10^{-10}$	Affective
Types of physical activity in last 4 weeks heavy DIY	-	0.000	$2.50x10^{-10}$	$1.20x10^{-01}$	307812	$9.40x10^{-57}$	Motor
Ever worried more than most people would in similar situation	+	0.001	$2.50x10^{-10}$	$1.07x10^{-02}$	72425	$2.19x10^{-11}$	Affective
Ever highly irritable argumentative for 2 days	+	0.001	$2.74x10^{-10}$	$1.33x10^{-01}$	99716	$5.39x10^{-09}$	Affective
Beef intake	-	0.000	$2.88x10^{-10}$	$1.22x10^{-01}$	306645	$2.50x10^{-04}$	Behavioural
FI3 word interpolation	-	0.000	$3.76x10^{-10}$	$4.36x10^{-03}$	98680	$3.72x10^{-04}$	Cognitive
Average weekly beer plus cider intake	+	0.000	$5.16x10^{-10}$	$1.42x10^{-01}$	224335	$3.94x10^{-08}$	Behavioural
Mood swings	+	0.000	$8.63x10^{-10}$	$3.55x10^{-04}$	300051	$4.78x10^{-29}$	Affective
Types of transport used excluding work walk	-	0.000	$1.22x10^{-09}$	$1.69x10^{-03}$	307812	$1.58x10^{-03}$	Behavioural
Transport type for commuting to job workplace car	-	0.000	$3.37x10^{-09}$	$2.15x10^{-01}$	307812	$3.01x10^{-194}$	Behavioural/motor
Victim of physically violent crime	+	0.000	$4.67x10^{-09}$	$7.91x10^{-02}$	83793	$5.37x10^{-03}$	Behavioural
Ever contemplated self-harm	+	0.000	$8.59x10^{-09}$	$1.15x10^{-01}$	83704	$1.76x10^{-07}$	Affective
Snoring	+	0.000	$1.21x10^{-08}$	$9.21x10^{-01}$	287640	$3.65x10^{-03}$	Behavioural/motor
Felt distant from other people in past month	+	0.001	$1.85x10^{-08}$	$1.71x10^{-01}$	31346	$1.58x10^{-04}$	Affective
Number of unsuccessful stop smoking attempts	+	0.000	$2.70x10^{-08}$	$3.50x10^{-02}$	68272	$1.39x10^{-01}$	Behavioural
Someone to take to doctor when needed as a child	-	0.000	$3.18x10^{-08}$	$3.04x10^{-02}$	83422	$1.30x10^{-01}$	Behavioural

Average total household income before tax	-	0.000	$3.33x10^{-08}$	$3.34x10^{-03}$	264483	$6.66x10^{-134}$	Behavioural
Physical violence by partner or ex-partner as an adult	+	0.000	$3.48x10^{-08}$	$5.85x10^{-01}$	83707	$6.27x10^{-01}$	Behavioural
Morning evening person chronotype	+	0.000	$3.76x10^{-08}$	$3.91x10^{-01}$	274298	$3.71x10^{-10}$	Motor/disorganisation
Health satisfaction	+	0.000	$3.99x10^{-08}$	$2.59x10^{-01}$	101574	$3.65x10^{-03}$	Disorganisation
Age at first episode of depression	-	0.001	$6.43x10^{-08}$	$2.84x10^{-01}$	32234	$4.91x10^{-06}$	Affective
Ever manic hyper for 2 days	+	0.001	$7.13x10^{-08}$	$5.45x10^{-02}$	99666	$1.31x10^{-25}$	Delusions
Recent worrying too much about different things	+	0.000	$8.48x10^{-08}$	$1.78x10^{-03}$	83578	$7.52x10^{-04}$	Affective
Frequency of walking for pleasure in last 4 weeks	+	0.000	$9.98x10^{-08}$	$2.80x10^{-03}$	222295	$4.40x10^{-04}$	Behavioural

GW-Sig PRS = PRS comprising only genome-wide significant (P_T<5x10^{-8}). Top non-overlapping and distinct traits in bold selected for Fig. 1 of the Main Text (*names given in Fig. 1 provided in parentheses if different*). The two last columns show the result (*P*-value) of a difference-in-means t-test between the target trait value among individuals diagnosed with schizophrenia and with unaffected individuals; and the traits categorised by schizophrenia symptom type respectively. $^{+}$ Fluid intelligence assessed by a simple 13 question verbal-numerical reasoning test.

Supplementary Table 3: Associations between self-reported risk-taking and measures of migration, substance use and control traits

	Effect (β): adjusted for age and sex	R^2: adjusted for age and sex	*P*-value: adjusted for age and sex	*P*-value: adjusted for age, sex and TDEP	*P*-value: adjusted for age, sex, TDEP and EDU
Ever smoked cannabis	0.23	0.11	$< 10^{-308}$	$< 10^{-308}$	$< 10^{-308}$
Time at current residence	-2.23	0.00664	$< 10^{-308}$	$< 10^{-308}$	$< 10^{-308}$
Left hand grip strength	0.651	0.000627	$1.32x10^{-143}$	$1.23x10^{-200}$	$9.95x10^{-181}$
Population density: current residence	204	0.00149	$3.19x10^{-136}$	$4.94x10^{-50}$	$7.28x10^{-24}$
Distance travelled: birth to current	10800	0.00131	$2.61x10^{-123}$	$1.14x10^{-133}$	$2.38x10^{-67}$
Self-reported substance abuse	1.85^{+}	0.00771^{α}	$7.94x10^{-74}$	$5.52x10^{-55}$	$3.93x10^{-62}$
Breast fed	1.16^{+}	0.00115^{α}	$4.67x10^{-67}$	$2.39x10^{-68}$	$5.95x10^{-51}$
Leg pain on walking	1.23^{+}	0.00229^{α}	$2.55x10^{-56}$	$3.85x10^{-38}$	$1.52x10^{-52}$
Population density difference: birth to current	127	0.000389	$7.13x10^{-37}$	$2.31x10^{-09}$	0.0252
Population density: birth place	76.8	0.000198	$1.50x10^{-19}$	$1.67x10^{-10}$	$2.44x10^{-10}$
Birth weight	0.0254	0.000271	$1.39x 10^{-17}$	$4.53x10^{-20}$	$1.45x10^{-16}$
Birth weight known	0.96^{+}	$9.45xl0^{-05\alpha}$	$2.80x10^{-09}$	0.000935	$4.39x10^{-05}$
Blood pressure device ID	156000	$2.50x10^{-06}$	0.277	0.267	0.331
Month attended baseline assessment	-0.00327	$1.87x10^{-07}$	0.766	0.778	0.86

+ Odds ratio for binary outcome variables; α Pseudo R^2 for binary outcome variables; TDEP = Townsend deprivation index; EDU = Educational attainment

Supplementary Table 4: Summary of PRS results corresponding to the most predictive PRS for schizophrenia tested against the target behavioural and control traits

	Effect (β): adjusted for age and sex	R^2: adjusted for age and sex	*P*-value: adjusted for age and sex	*P*-value: adjusted for age, sex and TDEP	*P*-value: adjusted for age, sex, TDEP and EDU
Time at current residence	-738.61	0.00027	$1.13x10^{-21}$	$2.32x10^{-21}$	$9.07x10^{-20}$
Population density: current residence	104222	$5.74x10^{-05}$	$3.28x10^{-11}$	$8.84x10^{-07}$	$8.65x10^{-06}$
Self-reported substance abuse	633.75^{+}	0.00168^{α}	$3.78x10^{-09}$	$8.75x10^{-09}$	$1.34x10^{-08}$
Ever smoked cannabis	13.04	0.00033	$7.01x10^{-08}$	$3.02x10^{-07}$	$1.17x10^{-06}$
Distance travelled: birth to current	-9246200	$9.32x10^{-06}$	0.0005	0.0008	0.0002
Blood pressure device ID	-670154	0.00014	0.0029	0.0029	0.0029
Left hand grip strength	-888.61	$1.15x10^{-05}$	0.0054	0.0421	0.0233
Population density difference: birth to current	43655.70	$2.11x10^{-05}$	0.0061	0.0500	0.1656
Breast fed	10.41^{+}	$2.08x10^{-05\alpha}$	0.0585	0.0432	0.0784
Population density: birth place	91061.90	$1.03x10^{-05}$	0.0673	0.1019	0.0609
Month attended baseline assessment	33.70	$8.32x10^{-06}$	0.0989	0.0885	0.0987
Leg pain on walking	113.19^{+}	$2.40x10^{-05\alpha}$	0.2134	0.3905	0.2845
Birth weight known	59.44^{+}	$2.88x10^{-06\alpha}$	0.3291	0.4623	0.3974
Birth weight	-4.56	$3.74x10^{-06}$	0.4134	0.4639	0.4058

+ Odds ratio for binary outcome variables; α Pseudo R^2 for binary outcome variables; TDEP = Townsend deprivation index; EDU = Educational attainment

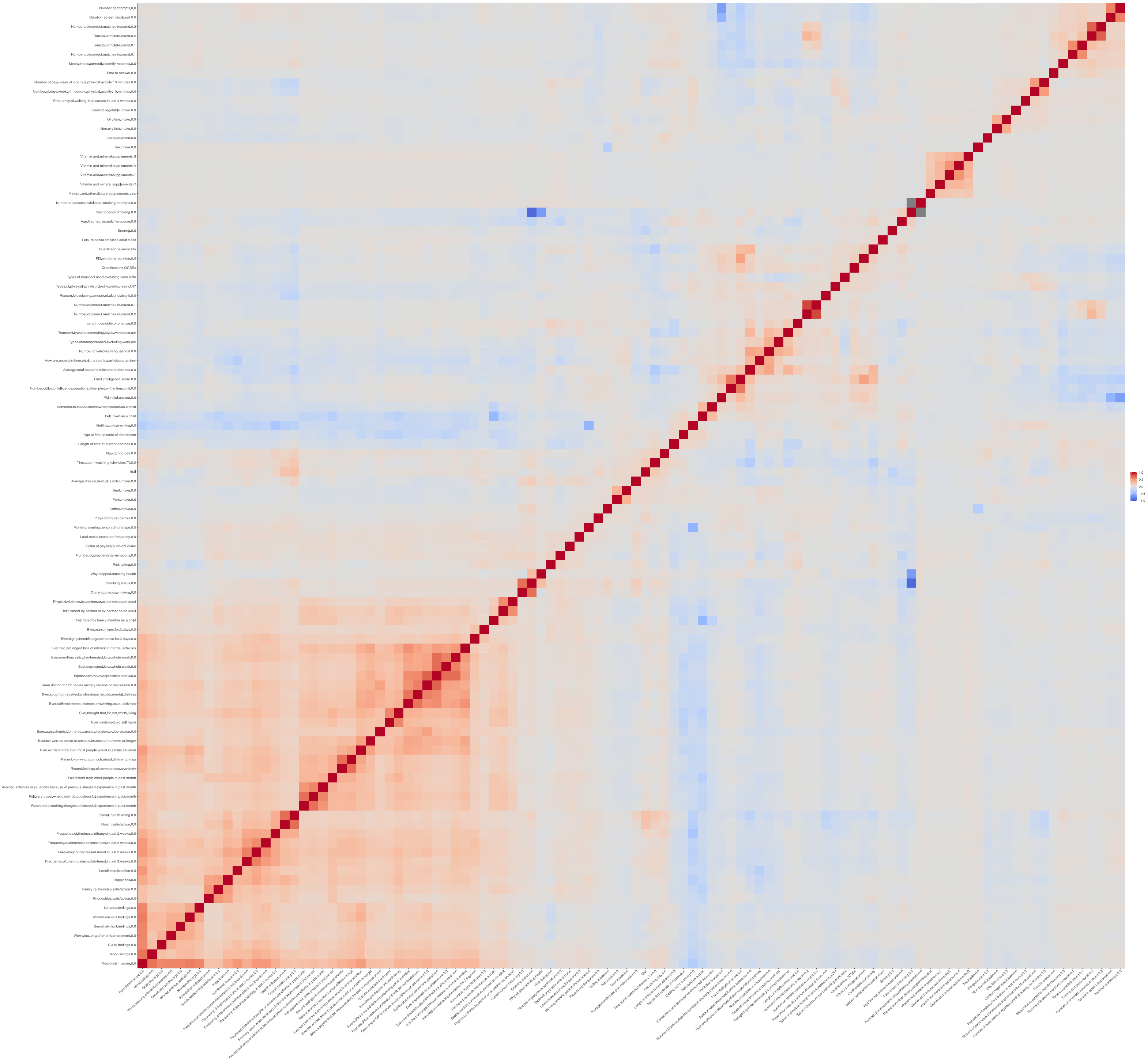

Supplementary Figure 1. Correlation matrix showing phenotypic correlations between the 104 target traits associated with schizophrenia PRS at *P*<1x10^{-7}. Not including same variable correlations, there are 10712 correlations in total, with a median r^2 of 0.01. 20% of the correlations are r^2>0.01; 6.8% are r^2>0.05; and 2.7% are r^2>0.1. This demonstrates overall weak intercorrelation between the behavioural traits included in the analysis.

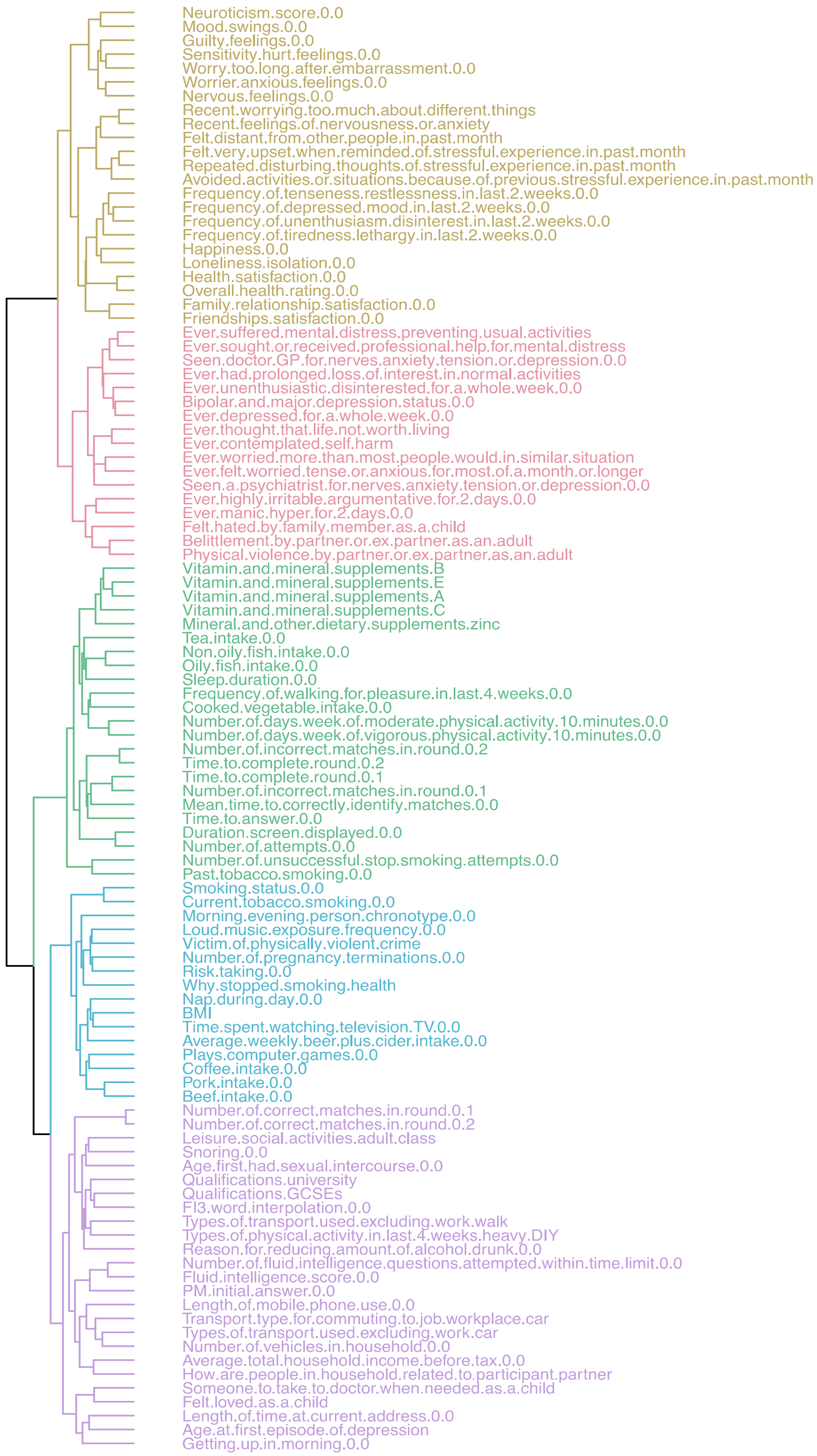

Supplementary Figure 2. Dendrogram showing phenotypic clustering based on the correlation matrix in Supplementary Fig. 2 for the 104 target traits associated with schizophrenia PRS at P<1x10^{-}

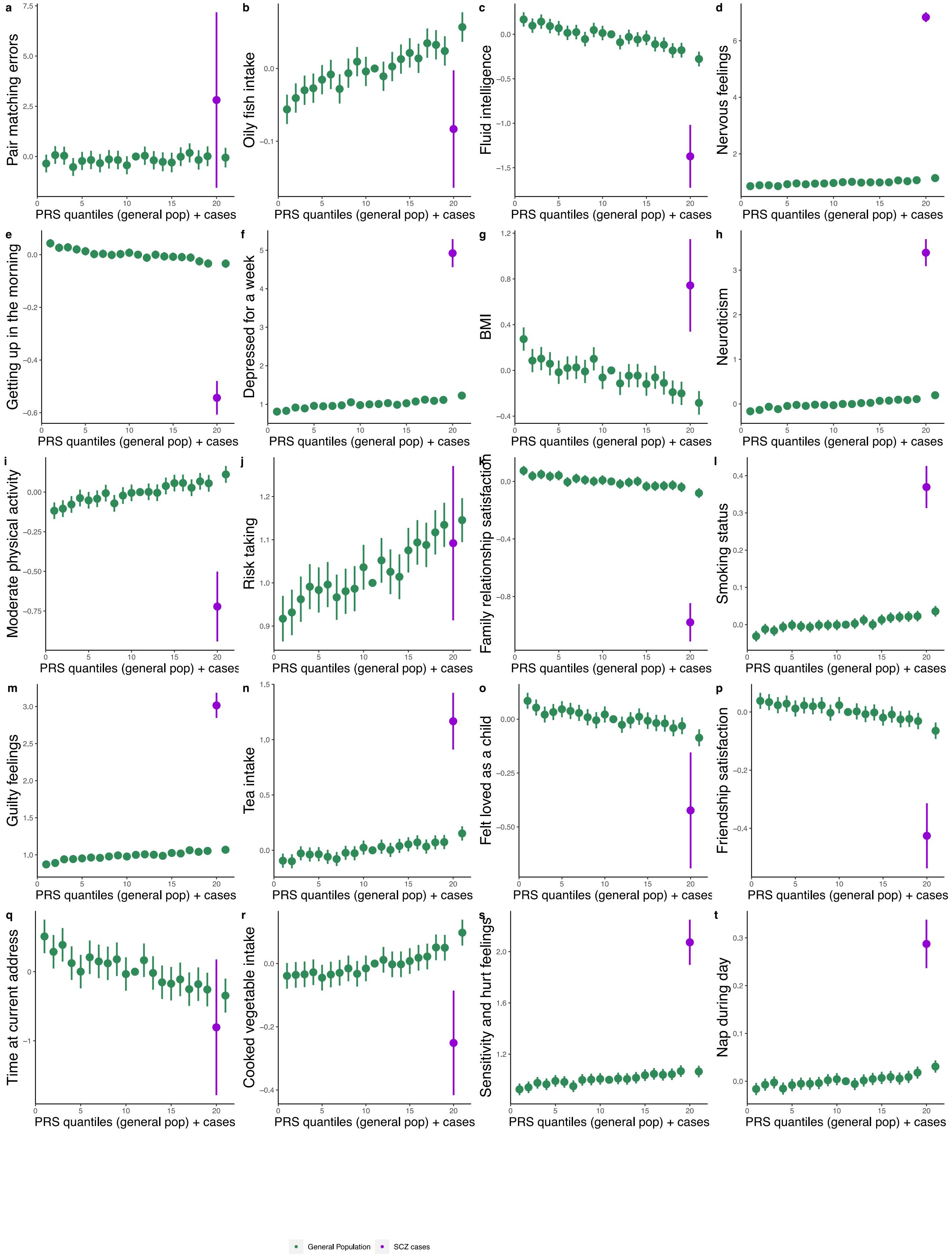

Supplementary Figure 3. PRS-by-trait quantile plots (in green) indicating the trends of association between the best-fit schizophrenia PRS and the target behavioural traits among 20 of the most significant associations, matching those of **Fig. 1**. The quantile plot is ordered from low to high genetic risk for schizophrenia, according to PRS in the target (UK Biobank) data, with each green point representing the average target trait value of a 5% quantile of the sample. Schizophrenia cases (purple) show the average PRS quantile of cases compared to the quantiles of the general population.

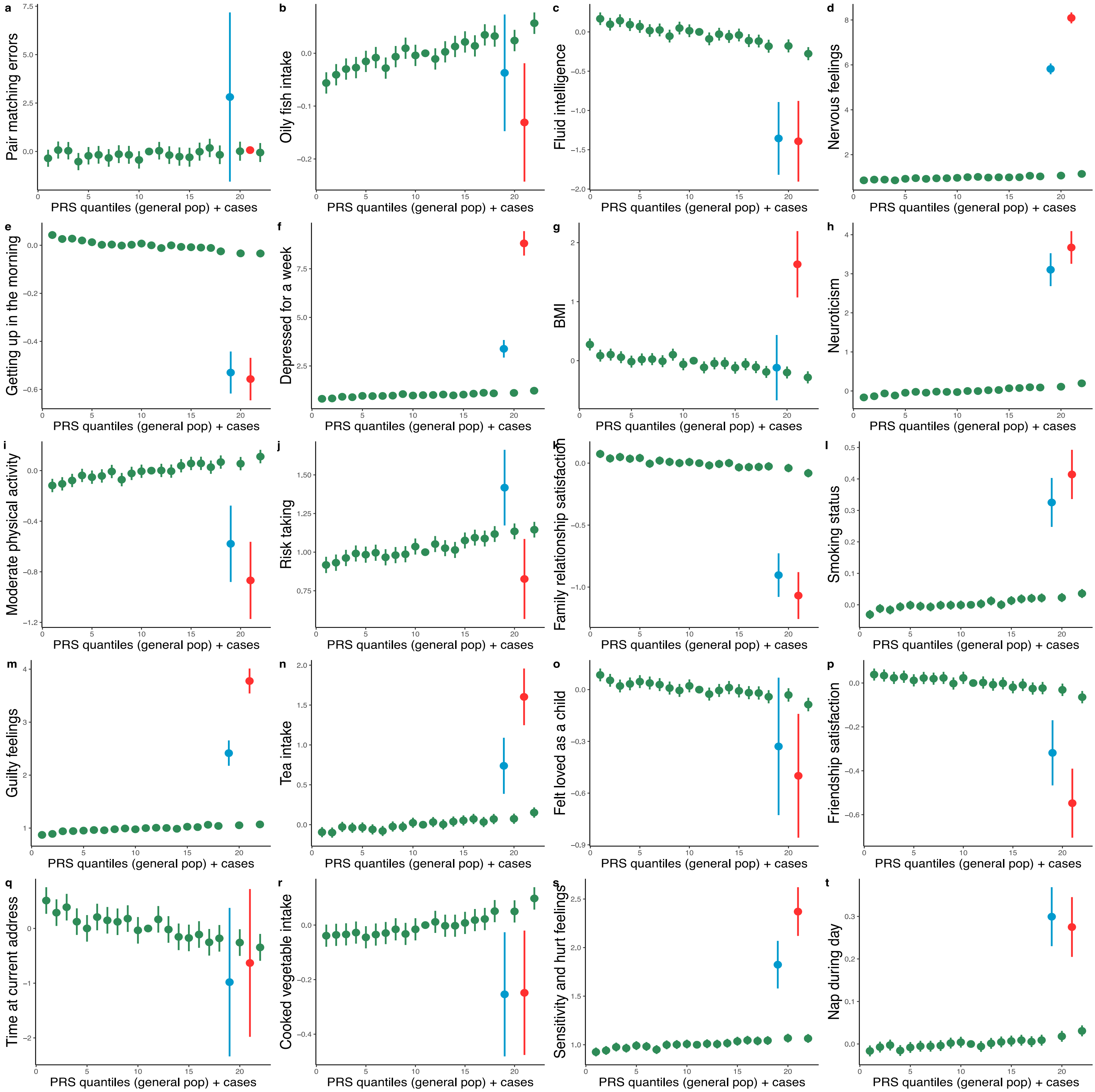

Supplementary

General Population (screened)
SCZ cases, non−medicated
SCZ cases, medicated

Figure 4. PRS-by-trait quantile plots (in green) indicating the trends of association between the best-fit schizophrenia PRS and the target behavioural traits among 20 of the most significant associations, matching those of **Fig. 1**. The quantile plot is ordered from low to high genetic risk for schizophrenia, according to PRS in the target (UK Biobank) data, with each green point representing the average target trait value of a 5% quantile of the sample. Medicated (red) and non-medicated (blue) schizophrenia cases show the average PRS quantile of medicated and non-medicated cases compared to the quantiles of the general population.

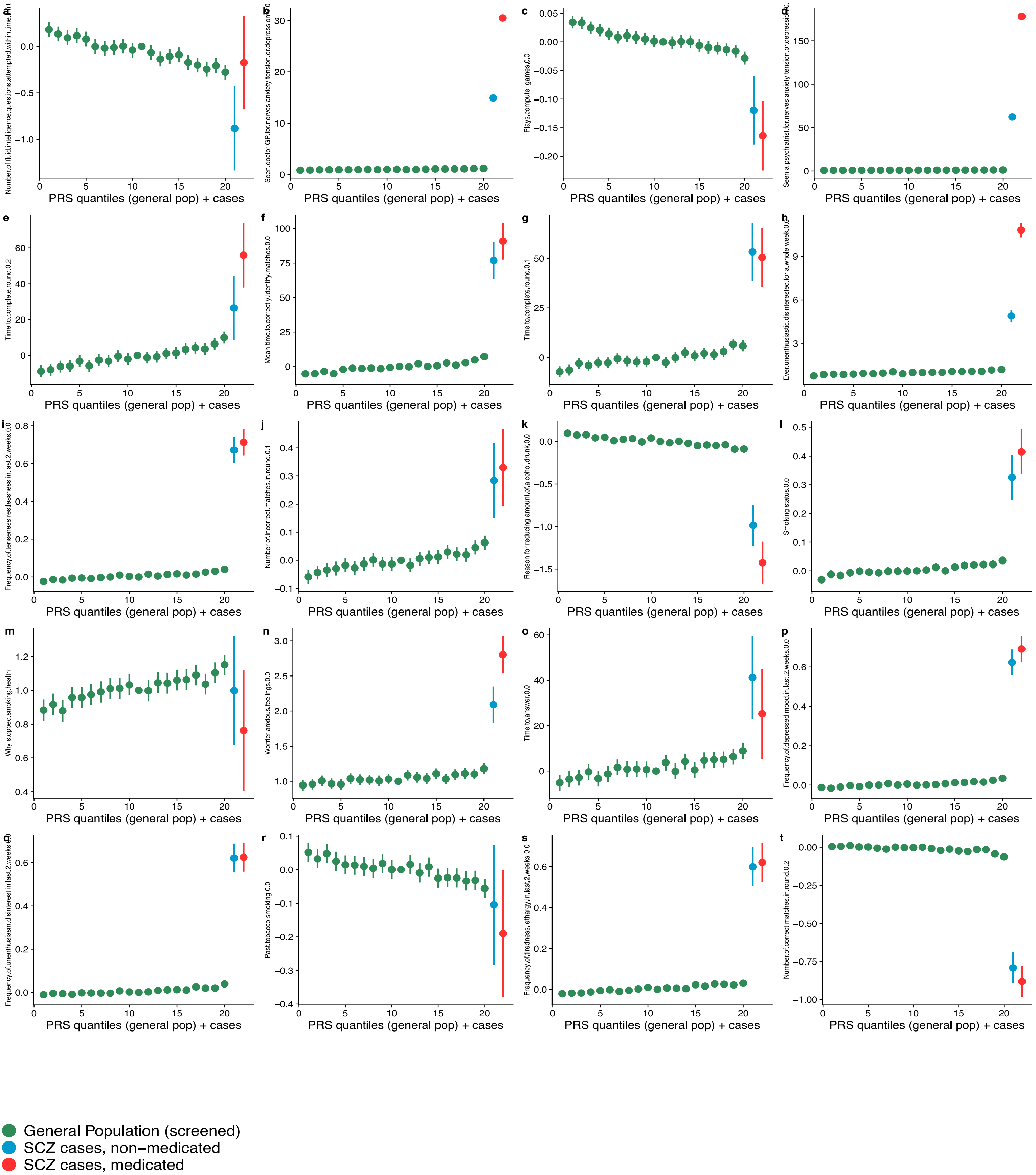

Supplementary Figure 5. PRS-by-trait quantile plots (in green) indicating the trends of association between the best-fit schizophrenia PRS and the target behavioural traits among all significant associations ($P < 1x10^{-7}$), ordered by significance, excluding those of Fig. 1 (see Supplementary Table 2 for details). Each quantile plot is ordered from low to high genetic risk for schizophrenia, according to PRS in the target (UK Biobank) data, with each green point representing the average target trait value of a 5% quantile of the sample. Non-medicated (blue) and medicated (red), at baseline, diagnosed individuals are appended to the right end of each plot, reflecting the expected higher genetic burden of diagnosed individuals compared to unaffected individuals (see Main Text). Vertical lines represent 95% confidence intervals; these appear absent for some traits with a large range and are larger in the two categories of cases due to their smaller sample sizes.

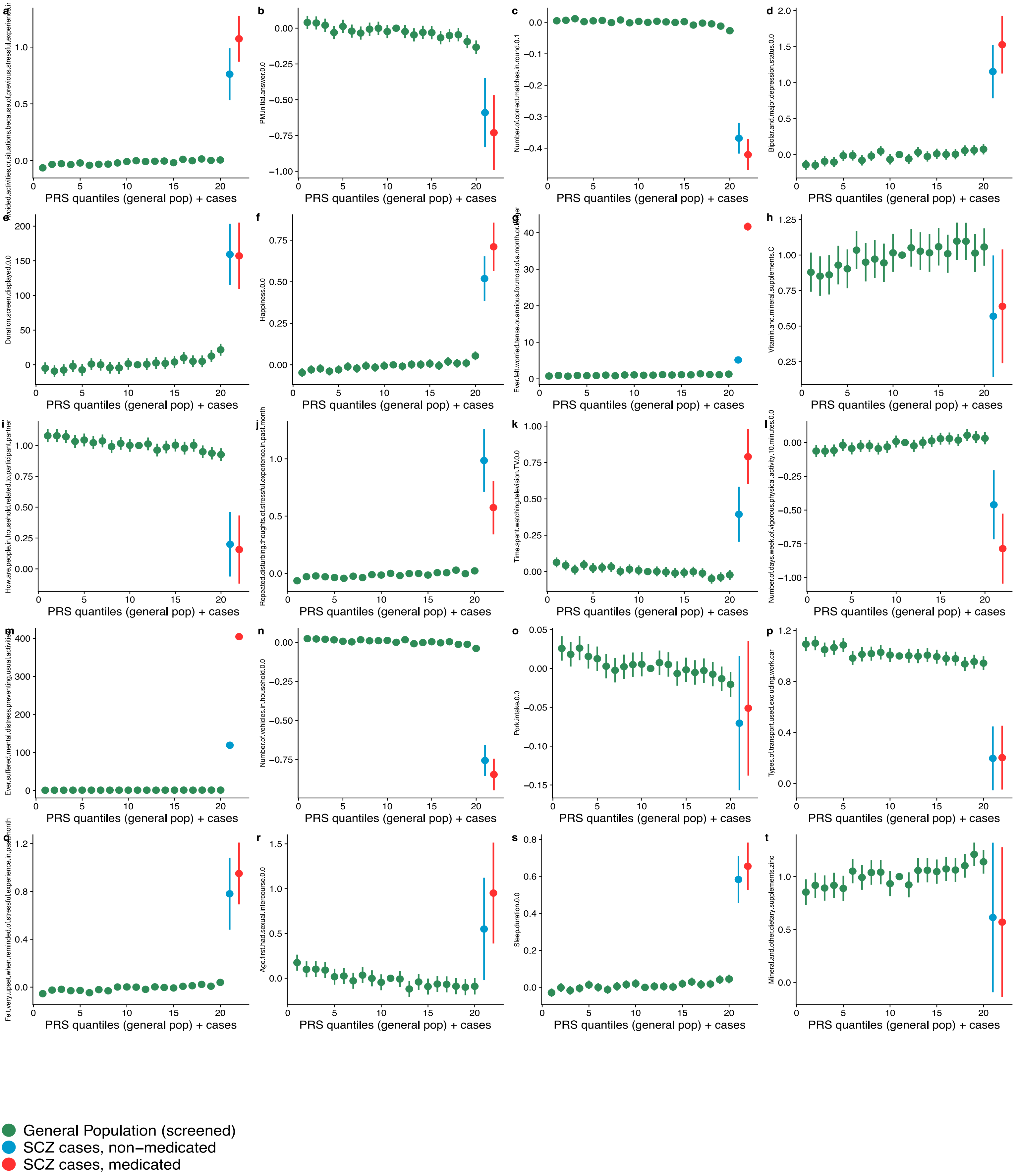

Supplementary Figure 6. PRS-by-trait quantile plots (in green) indicating the trends of association between the best-fit schizophrenia PRS and the target behavioural traits among all significant associations ($P < 1x10^{-7}$), ordered by significance, excluding those of Fig. 1 (see Supplementary Table 2 for details). Each quantile plot is ordered from low to high genetic risk for schizophrenia, according to PRS in the target (UK Biobank) data, with each green point representing the average target trait value of a 5% quantile of the sample. Non-medicated (blue) and medicated (red), at baseline, diagnosed individuals are appended to the right end of each plot, reflecting the expected higher genetic burden of diagnosed individuals compared to unaffected individuals (see Main Text). Vertical lines represent 95% confidence intervals; these appear absent for some traits with a large range and are larger in the two categories of cases due to their smaller sample sizes.

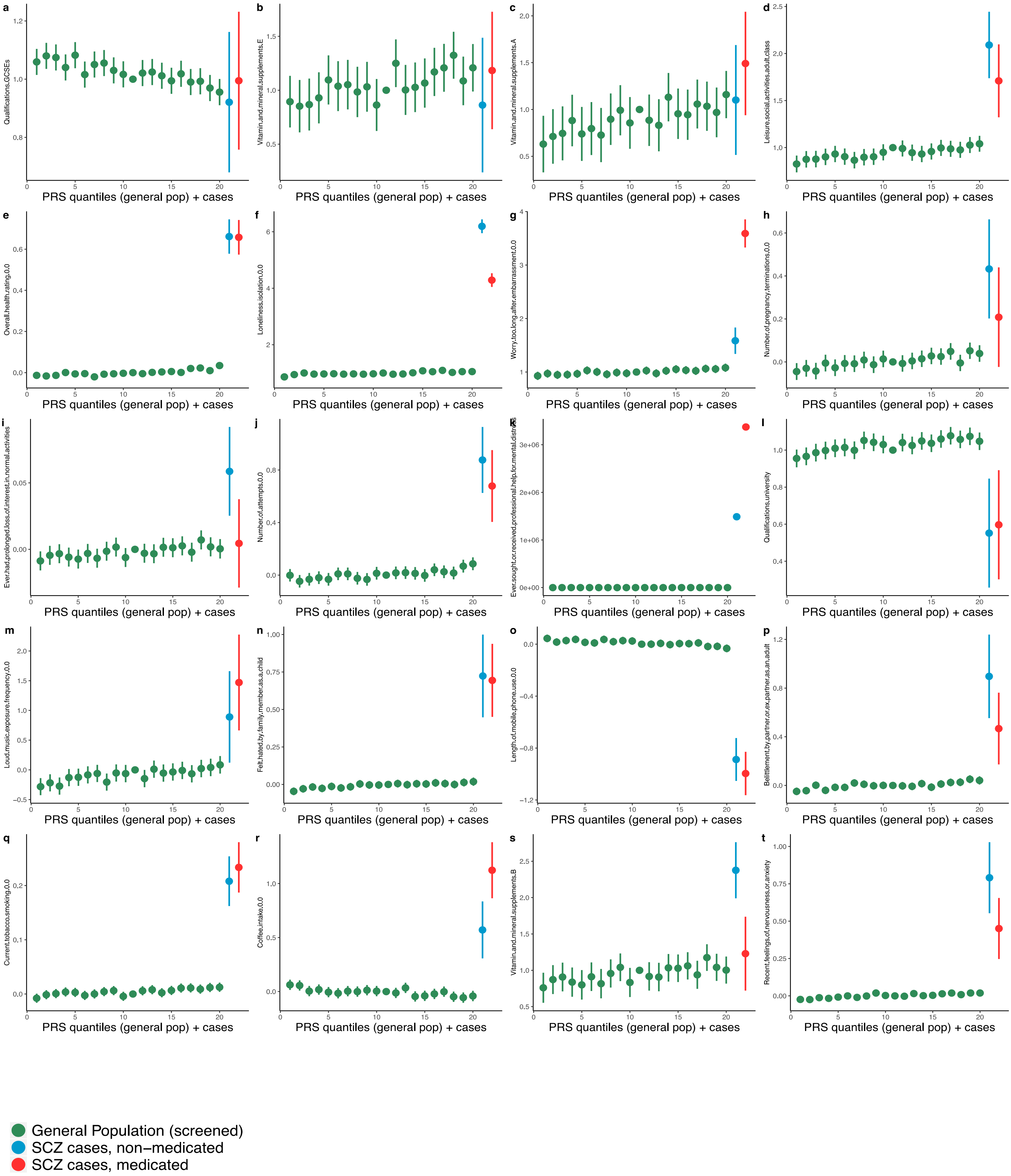

Supplementary Figure 7. PRS-by-trait quantile plots (in green) indicating the trends of association between the best-fit schizophrenia PRS and the target behavioural traits among all significant associations (*P* < $1x10^{-7}$), ordered by significance, excluding those of Fig. 1 (see Supplementary Table 2 for details). Each quantile plot is ordered from low to high genetic risk for schizophrenia, according to PRS in the target (UK Biobank) data, with each green point representing the average target trait value of a 5% quantile of the sample. Non-medicated (blue) and medicated (red), at baseline, diagnosed individuals are appended to the right end of each plot, reflecting the expected higher genetic burden of diagnosed individuals compared to unaffected individuals (see Main Text). Vertical lines represent 95% confidence intervals; these appear absent for some traits with a large range and are larger in the two categories of cases due to their smaller sample sizes.

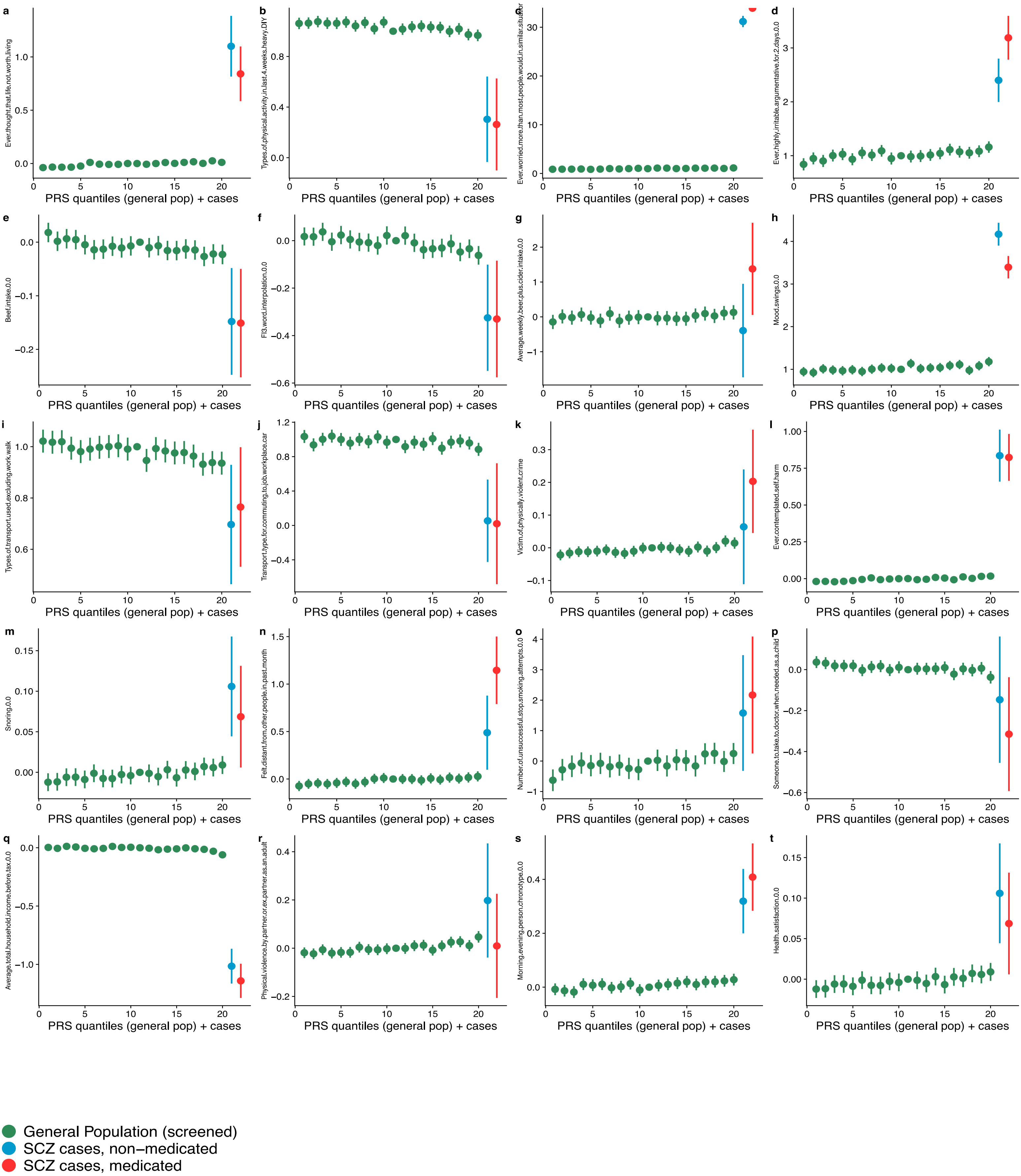

Supplementary Figure 8. PRS-by-trait quantile plots (in green) indicating the trends of association between the best-fit schizophrenia PRS and the target behavioural traits among all significant associations ($P < 1x10^{-7}$), ordered by significance, excluding those of Fig. 1 (see Supplementary Table 2 for details). Each quantile plot is ordered from low to high genetic risk for schizophrenia, according to PRS in the target (UK Biobank) data, with each green point representing the average target trait value of a 5% quantile of the sample. Non-medicated (blue) and medicated (red), at baseline, diagnosed individuals are appended to the right end of each plot, reflecting the expected higher genetic burden of diagnosed individuals compared to unaffected individuals (see Main Text). Vertical lines represent 95% confidence intervals; these appear absent for some traits with a large range and are larger in the two categories of cases due to their smaller sample sizes.

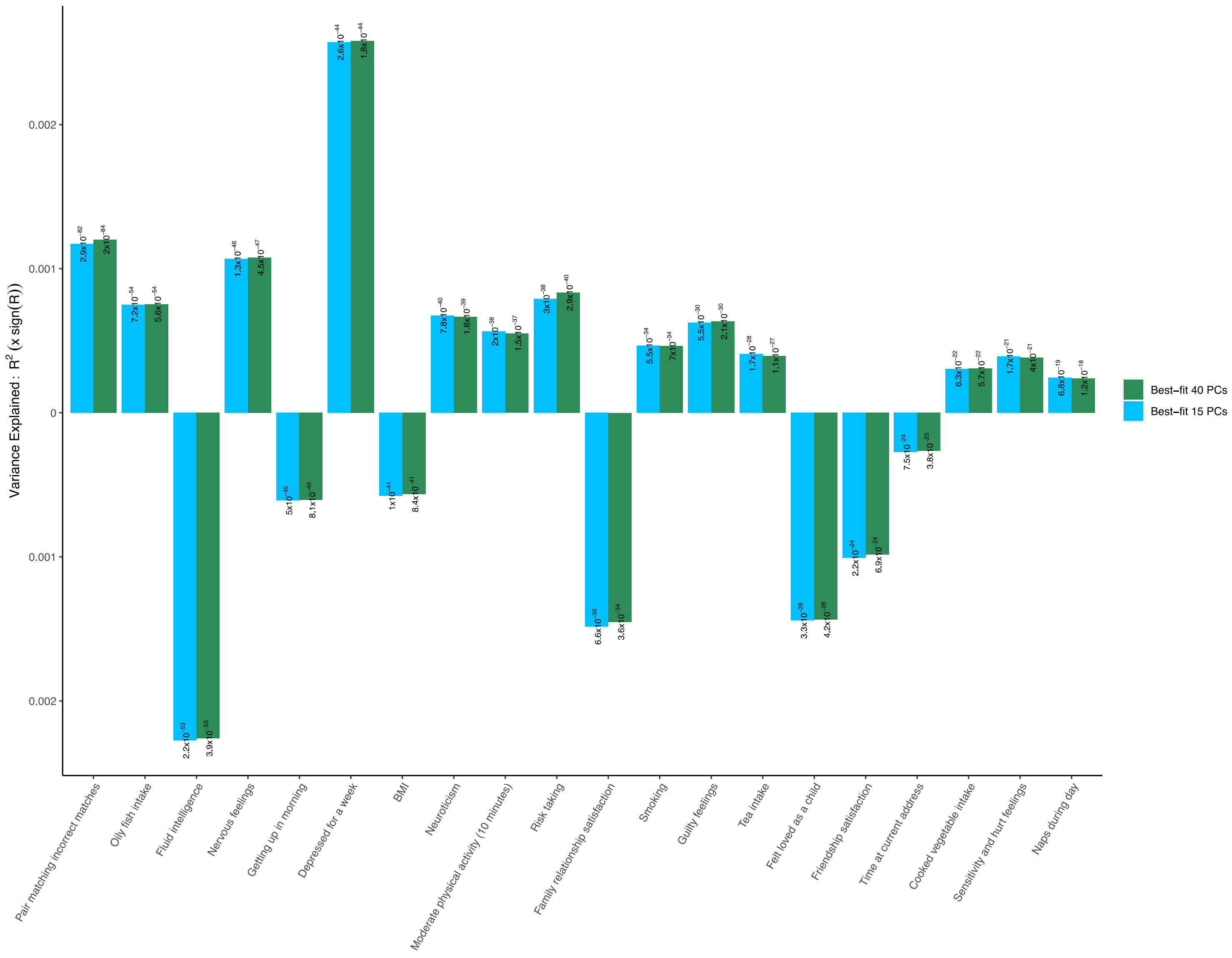

Supplementary Figure 9. A comparison between the results corresponding to Fig. 1 adjusting for 15 principal components (PCs), in green, and adjusting for 40 PCs, in blue.

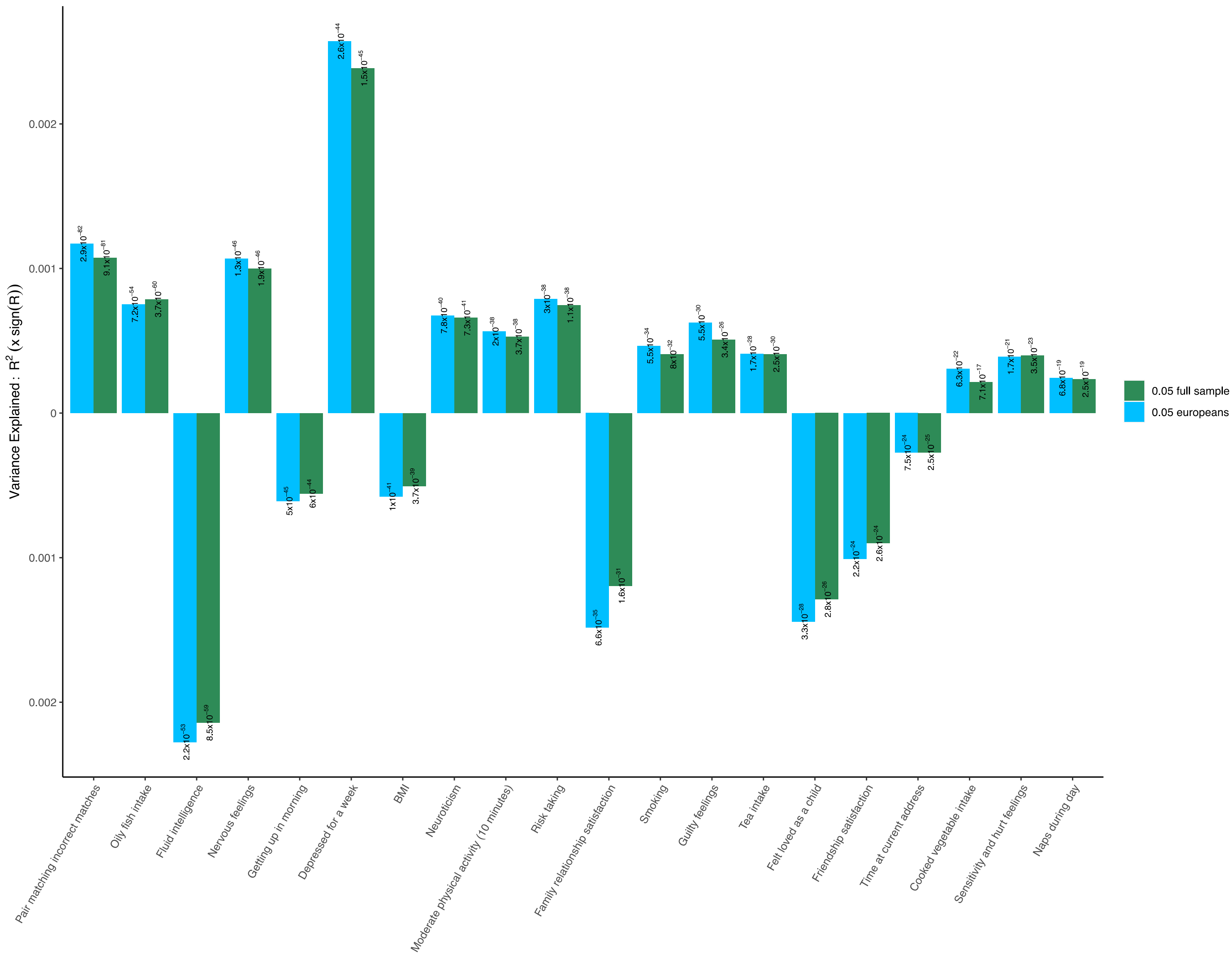

Supplementary Figure 10. A comparison between the results corresponding to Fig. 1 showing the Europeans only sample, in green, and the full transethnic sample, in blue.
